# The phagocytic cyst cells in *Drosophila* testis eliminate germ cell progenitors via phagoptosis

**DOI:** 10.1126/sciadv.abm4937

**Published:** 2022-06-17

**Authors:** Maayan Zohar-Fux, Aya Ben-Hamo-Arad, Tal Arad, Marina Volin, Boris Shklyar, Ketty Hakim-Mishnaevski, Lilach Porat-Kuperstein, Estee Kurant, Hila Toledano

**Affiliations:** 1 Department of Human Biology, Faculty of Natural Sciences, University of Haifa, 199 Aba Hushi Avenue, Mount Carmel, Haifa 3498838, Israel.; 2 Bioimaging Unit, Faculty of Natural Sciences, University of Haifa, 199 Aba Hushi Avenue, Mount Carmel, Haifa 3498838, Israel.

## Abstract

Phagoptosis is a frequently occurring nonautonomous cell death pathway in which phagocytes eliminate viable cells. While it is thought that phosphatidylserine (PS) “eat-me” signals on target cells initiate the process, the precise sequence of events is largely unknown. Here, we show that in *Drosophila* testes, progenitor germ cells are spontaneously removed by neighboring cyst cells through phagoptosis. Using live imaging with multiple markers, we demonstrate that cyst cell–derived early/late endosomes and lysosomes fused around live progenitors to acidify them, before DNA fragmentation and substantial PS exposure on the germ cell surface. Furthermore, the phagocytic receptor Draper is expressed on cyst cell membranes and is necessary for phagoptosis. Significantly, germ cell death is blocked by knockdown of either the endosomal component Rab5 or the lysosomal associated protein Lamp1, within the cyst cells. These data ascribe an active role for phagocytic cyst cells in removal of live germ cell progenitors.

## Teaser

During spermatogenesis, phagocytes execute excessive live germ cell progenitors without any evidential provocation.

## INTRODUCTION

Programmed cell death (PCD) functions to remove surplus or damaged cells throughout development and during tissue homeostasis of adult organisms (*1*). There are several types of PCD, the most commonly studied of which are apoptosis, autophagy-dependent cell death, and programmed necrosis, all of which are regarded as autonomous cell decisions to commit suicide. The resulting cell debris or apoptotic bodies are removed by phagocytes that are recruited to engulf the dead/dying cell, via recognition of “eat-me” signals, such as phosphatidylserine (PS), exposed on the cell surface. Following engulfment, lysosomal activity is induced to degrade the internalized content (*2*, *3*). According to this paradigm, only dead cells or cells doomed to die are eliminated by phagocytosis. However, a newly identified type of PCD has been described, named phagoptosis, in which phagocytes engulf and degrade viable cells, thereby leading to their elimination in a cell nonautonomous manner (*4*, *5*). By definition, inhibition of the phagocytic machinery within phagocytes prevents death of the target cells. Although relatively recently recognized, phagoptosis is the most prevalent type of PCD in adult tissues, as it mediates the continual removal of short-lived blood cells, including erythrocytes (*6*) and neutrophils (*7*). Activation of neutrophils by pathogens is accompanied by PS exposure on the surface of the activated but viable cells, leading to their phagoptosis. Moreover, old erythrocytes down-regulate CD47, which is expressed on the surface of young cells and acts as a “don’t-eat-me” signal to block phagoptosis (*8*). Hence, it was suggested that phagoptosis is induced by the reversible exposure of PS or other eat-me signals, or by the loss of don’t-eat-me protection signals, on the outer leaflet of an otherwise viable cell. Challenging this premise, however, was the finding that elevated expression of phagocytic receptors in adult *Drosophila* glia or in the ovary was sufficient to induce phagoptosis of live neurons and live germ cells, respectively (*9*, *10*). It is not known whether phagocytes can induce phagoptosis without prior signals from the target cells under homeostatic conditions.

In the mammalian testis, PCD occurs during early stages of transit-amplified progenitors, before the cells reach terminal differentiation. This generates a mass of cells that must be effectively removed from the inner compartment of the testis (*11*, *12*). In notable resemblance to mammals, as many as a quarter of the newly emerging spermatogonia progenitors in the *Drosophila* testis are spontaneously eliminated by germ cell death (GCD) (*12*–*14*). As transit-amplified progenitors develop and die as an interconnected “cyst” comprising 2 to 16 germ cells, debris is often large and composed of clumps of dying cells (*15*, *16*). GCD markedly increases during prolonged protein starvation to provide nutrients and protect the germline stem cell (GSC) population. Under these stress conditions, one of the two cyst cells that encapsulate the spermatogonia dies by apoptosis, which then triggers GCD, whereas the other cyst cell clears the resulting debris (*17*, *18*). However, the mechanism of GCD under homeostasis remains elusive. It is known that GCD does not involve the classic apoptotic machinery, occurring in the absence of the main apoptotic effector caspases and the apoptosome. GCD is associated with chromatin condensation, chromosomal DNA fragmentation, and high lysosomal activity (*12*). However, genetic screens revealed no role for macroautophagy in GCD, even though lysosomes are activated.

In this study, we show that live progenitors die by phagoptosis, during which neighboring cyst cells generate early and late endosomes and then lysosomes, which fuse around live spermatogonia, leading first to acidification within the germ cells, followed later by DNA fragmentation and degradation. Unexpectedly, lysosomal activity is detected before PS exposure, which is only observed at later stages of degradation. Blocking the phagocytic machinery within cyst cells arrests GCD. We further show that the transmembrane phagocytic receptor Draper (Drpr) (*19*), the *Drosophila* ortholog of mammalian Jedi/LRP/MEGF10 (*20*, *21*), is expressed in cyst cells and is required to mediate phagoptosis of germ cell progenitors that would otherwise have remained viable. Together, our research reveals that phagocytic cyst cells execute germ cells in a cell nonautonomous manner, which is critical for normal spermatogenesis.

## RESULTS

### Lysosomal activity precedes DNA fragmentation in dying germ cells

**Fig. 1.**
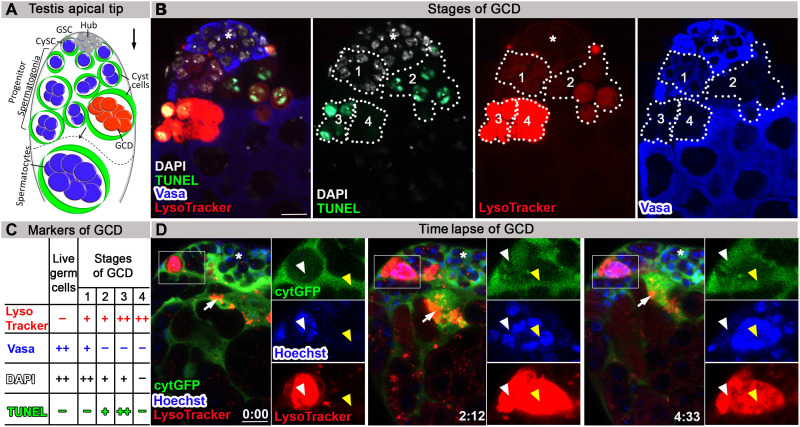
Lysosomal activity precedes DNA fragmentation in dying germ cells. (**A**) Schematic representation of the apical tip of the testis (side view). GSCs (blue) intermingle with cyst stem cells (CySCs; green) around the hub (gray). Spermatogonia germ cells (blue) are transit-amplified progenitor cells encapsulated by cyst cells (green). About one-quarter of spermatogonia undergo GCD (red). The dashed line separates spermatogonia from terminally differentiated spermatocytes. The differentiation axis (arrow) runs from the apical (stem cell niche) to the basal end (sperm maturation). (**B**) Representative wild-type testis (*w1118*, 2 days old, *n* = 49) immunostained for TUNEL (green, fragmented DNA), DAPI (white, DNA), LysoTracker (red, lysosomal activity), and Vasa (blue, germ cells). Different combination of the markers appears in four stages of GCD progression. Note the lysosomal activity at stage 1 without TUNEL and with Vasa staining. (**C**) Summary of the markers that appear in live germ cells and in the four stages of GCD. (**D**) Snapshots of live-imaged testis, marked with LysoTracker (red), Hoechst (blue, nuclei), and GFP (cyst cells, *c587Gal4;UAS-cytGFP*). Time (hour:min) is shown on the bottom middle of the images. Note rectangles and blown-up insets highlighting two adjacent GCD events, marked with yellow and white arrowheads. White arrowheads mark GCD event that is completely degraded within a cyst cell within ~2 hours. Yellow arrowheads mark GCD event that begins after ~2 hours with the onset of LysoTracker, depicting packed DNA in separate nuclei that are further involuted into one bundle. Arrows mark cyst cell containing LysoTracker-positive debris, asterisks mark the hub, and scale bars correspond to 10 μm.

In nearly all adult *Drosophila* testes, one to several GCD events of spermatogonia occur spontaneously at the apical tip (a schematic is illustrated in Fig. 1A). To study the process of GCD and to determine the sequence of events, the combination of four immunofluorescent markers was applied: (i) TUNEL (terminal deoxynucleotidyl transferase–mediated deoxyuridine triphosphate nick end labeling) to detect fragmented DNA in dying cells, (ii) LysoTracker to track acidification within cellular compartments, namely, lysosomes, (iii) anti-Vasa antibodies to mark live germ cells, and (iv) 4′,6-diamidino-2-phenylindole (DAPI) staining to visualize the nuclei. As expected, live germ cells marked with strong DAPI and Vasa staining were detected at the apical tip. In addition, up to four distinct GCD events were evident, likely representing distinct stages of GCD progression (*n* = 49; Fig. 1, B and C). Figure 1B shows one sample in which all four GCD events were evident. In the first one, the DNA was intact (strong DAPI and no TUNEL staining); however, LysoTracker signal was observed. Vasa signal was detectable yet much weaker compared with live germ cells (Fig. 1B and fig. S1A), consistent with degradation of the cell’s protein content (*17*). At the second stage, LysoTracker and weak TUNEL signals were detected, and both increased in intensity at the third stage. At the fourth stage, LysoTracker signal remained strong, but there was no longer evidence of DNA (no DAPI). These data demonstrate that in GCD, lysosomal activity precedes DNA fragmentation (Fig. 1, B and C, and fig. S1A). To verify this sequence of GCD progression, live imaging was performed. During the live imaging (*n* = 4), the testes were maintained in media containing low concentration of LysoTracker and Hoechst to visualize in situ changes in lysosomal activity and DNA, respectively. The cyst cells that surround the germ cells were marked with cytoplasmic green fluorescent protein (cytGFP). At the onset, LysoTracker-positive cells contained packed DNA of each of the single germ cells that comprised the dying spermatogonia. As the death process progressed, the chromatin disintegrated, mixed into one bundle, and then degraded, supporting our findings that lysosomal activity occurs before DNA fragmentation (Fig. 1D and movie S1). According to the live imaging, DNA degradation takes an estimated 2 to 4 hours; however, the cell acidification that was detected by LysoTracker before DNA disintegration was maintained much longer. In addition, live and dying germ cell spermatogonia were surrounded by cyst cells, marked by cytGFP, which often colocalized with LysoTracker-positive debris (Fig. 1D and movie S1). On the basis of this observation and on the finding that lysosomal activity is one of the first events observed in GCD, we postulated that cyst cells may phagoptose live germ cells.

### Cyst cell–derived Lamp1 phagosomes are generated before germ cell acidification

**Fig. 2.**
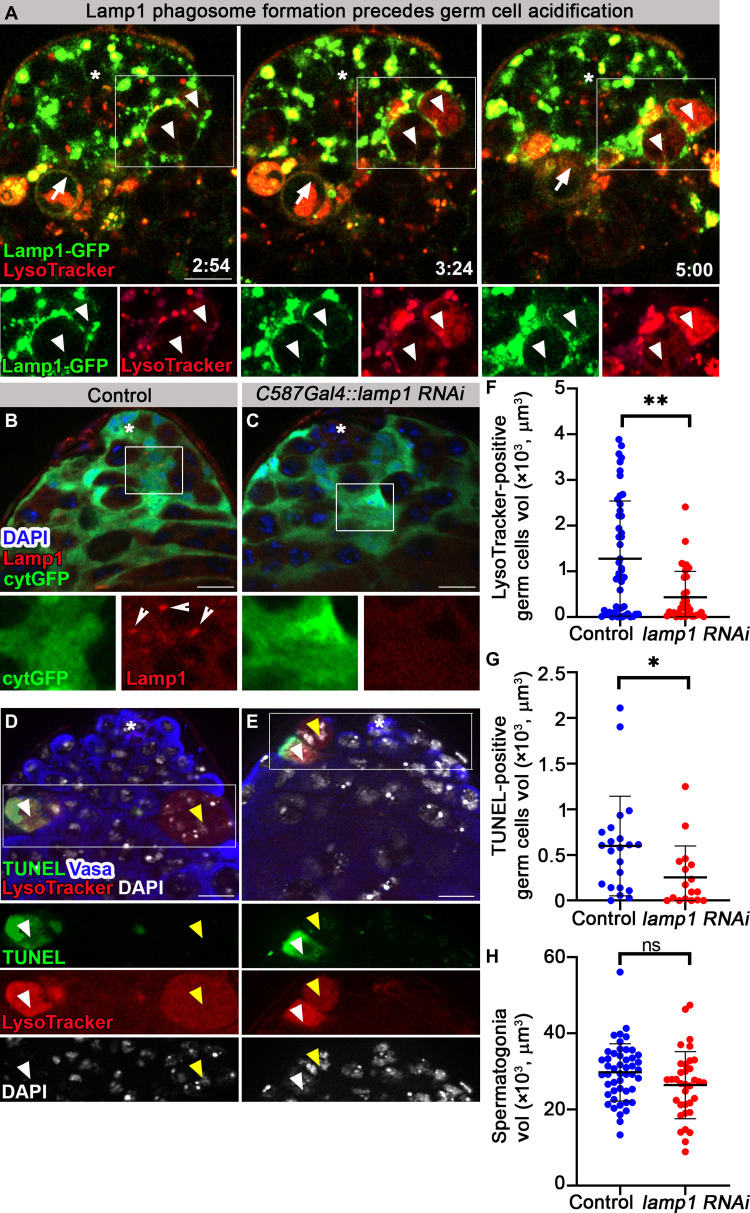
Cyst cell–derived Lamp1-containing lysosomes acidify germ cells. (**A**) Snapshots of live-imaged testis expressing Lamp1-GFP in cyst cells (green, *c587Gal4;UAS-lamp1-GFP*) and marked with LysoTracker (red). Time (hour:min) is shown on the bottom right of the images. Bottom images (single-channel views of the boxed regions) highlight Lamp1-GFP–positive phagosomes around two adjacent live spermatogonia (white arrowheads) that are gradually filled with LysoTracker. A phagosome (arrow) containing LysoTracker-positive debris is faded away after ~5 hours, when debris is degraded. (**B** and **C**) Immunofluorescence images of testes from control flies expressing cytGFP in cyst cells [(B) *c587Gal4;UAS-cytGFP*] and a *lamp1 RNAi* transgene [(C) *c587Gal4;UAS-cytGFP,UAS- lamp1 RNAi*] stained for Lamp1 (red) and DAPI (blue). Bottom images are high-magnification views of the boxed regions, highlighting Lamp1 expression in cyst cells of control [(B) arrowheads] and *lamp1 RNAi* (C). (**D** and **E**) Testes of control [(D) *c587Gal4* outcrossed to *w1118*, *n* = 48] and a *lamp1 RNAi* transgene expressed in cyst cells [(E) *c587-Gal4,UAS- lamp1 RNAi*, *n* = 35] were stained for Vasa and Fas3 (blue, germ and hub cells), TUNEL (green), LysoTracker (red), and DAPI (white). Bottom images (single-channel views of the boxed regions) highlighting GCD events at stage 1, positive for LysoTracker and DAPI but negative for TUNEL (yellow arrowheads), or positive for LysoTracker and TUNEL and negative for DAPI (white arrowheads). (**F** to **H**) Quantification of the volume of LysoTracker-positive germ cells (F), TUNEL-positive germ cells (G), and live spermatogonia cells (H) as measured with Imaris (control, blue dots; and *lamp1 RNAi*, red dots). Note the significant reduction in GCD in testes of *lamp1 RNAi*–expressing flies (F and G) and no significant change in spermatogonia volume (H). Statistical significance was determined by a Mann-Whitney test, *P ≤ 0.05, **P ≤ 0.01. ns, not significant. Asterisks mark the hub, and scale bars correspond to 10 μm.

A key feature of phagoptosis is that it is a nonautonomous cell death. Therefore, if cyst cells phagoptose germ cells, their phagocytic machinery is expected to mediate the process. To assess this hypothesis, a GFP-tagged form of Lamp1 (Lamp1-GFP), one of the most abundant lysosomal membrane proteins, was expressed in cyst cells (*22*). In contrast to testes of control males (*n* = 16; fig. S2A), in Lamp1-GFP flies, Lamp1 localized in a punctate pattern with Armadillo, a cyst cell marker (*n* = 23; fig. S2B). Live imaging (*n* = 3) revealed Lamp1-GFP–positive lysosomes within cyst cells incorporating into phagosomes. Lamp1-containing phagosomes were detected to form de novo around spermatogonia germ cells, which gradually stained positive with LysoTracker (Fig. 2A and movie S2). These results suggest that the contents of lysosomes derived from cyst cells invade germ cells. To assess the contribution of cyst cell–derived lysosomes to GCD, RNA interference (RNAi)–mediated knockdown of *lamp1* was induced specifically within the cyst cells. Immunostaining the testes with anti-Lamp1 antibodies (*23*) revealed a punctate expression within cyst cells of control flies marked with cytGFP that was reduced in testes from *lamp1* RNAi (Fig. 2, B and C). The knockdown was further visualized by use of a Lamp1 biomarker, in which the mCherry tag was inserted at its endogenous chromosomal locus (*24*), thus marking Lamp1 expression in cyst cells (fig. S2C). Although the RNAi construct only partially reduced mCherry levels (fig. S2, C and D), it was sufficient to significantly reduce the volume of LysoTracker or TUNEL-positive germ cells by 2.8- and 2.6-fold, respectively (Fig. 2, D to G). Notably, although we found LysoTracker-positive, TUNEL-negative debris (stage 1 of GCD) in control and lamp RNAi genotypes, in none did we find TUNEL staining without LysoTracker staining. These results indicate that death did not occur in the absence of acidification (Fig. 2, D and E). Next, we measured also the volume of live spermatogonia to determine whether germ cells escaped from engulfment. Notably, compared with control, *lamp1* RNAi did not affect the volume of live spermatogonia (Fig. 2H), suggesting that, as expected, Lamp1 is required for acidification and not for engulfment. Together, these results support a nonautonomous role for cyst cells in phagoptosis of live germ cells.

### Early and late endosome formation precedes GCD

**Fig. 3.**
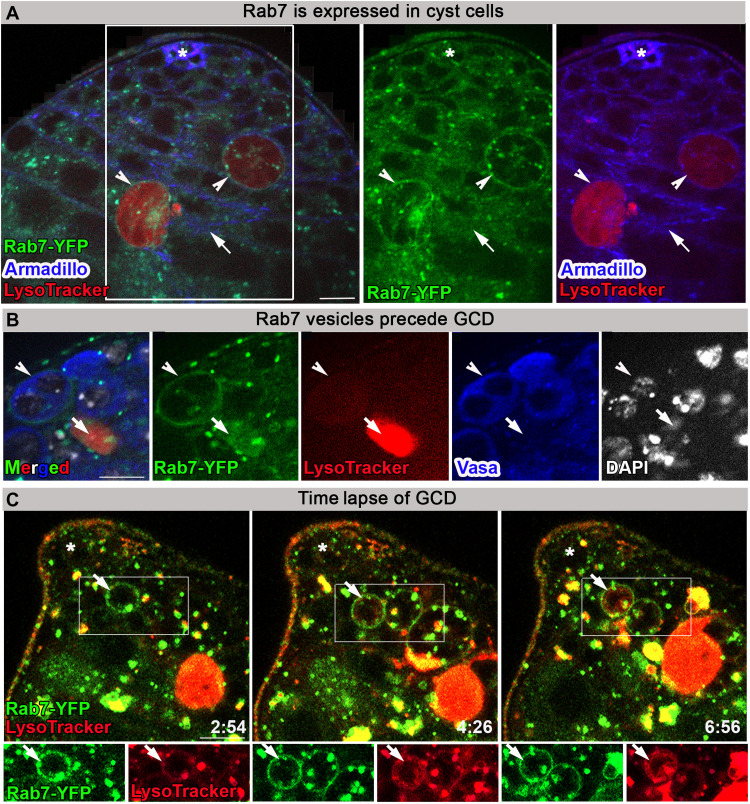
Rab7-containing late endosomes appear before GCD. (**A**) Immunofluorescence images of testis from flies expressing YFP at endogenous chromosomal locus of *rab7* (green, Rab7-YFP) labeled with LysoTracker (red) and Armadillo (blue, cyst and hub cells). Arrow marks Rab7 expression in cyst cells. Arrowheads mark Rab7 expression around dying germ cells. (**B**) Immunofluorescence images of Rab7-YFP (green) testis labeled with LysoTracker (red), Vasa (blue, live germ cells), and DAPI (white). Arrowhead marks Rab7-YFP phagosome around live germ cells expressing Vasa, and arrow marks germ cell debris with strong LysoTracker signal and no Vasa staining. (**C**) Snapshots of live-imaged testis from Rab7-YFP (green) marked with LysoTracker (red). Time (hour:min) is shown on the bottom right of the images. Bottom images are high-magnification views of the boxed regions, highlighting late endosomes (arrow) surrounding live germ cells that are gradually filled with LysoTracker. Asterisks mark the hub, and scale bars correspond to 10 μm.

During phagocytosis of apoptotic cells, Lamp1-containing vesicles fuse with phagosomes to form phagolysosomes that target the internalized content for degradation. This occurs following phagosome formation and fusion with early and late endosomes (*25*). As in vertebrates, Rab5 and Rab7 are Ras-related guanosine triphosphatases (GTPases) known to be associated with early and late endosomes, respectively. To follow Rab5- and Rab7-containing endosomes during GCD, yellow fluorescent protein (YFP) tags were inserted at their endogenous chromosomal loci (*26*). Rab7-YFP colocalized with Armadillo in cyst cells but also accumulated around dying germ cells (Fig. 3A). Immunostaining the testes of Rab7-YFP–expressing flies with Vasa revealed late endosomes around live germ cells before Vasa degradation and DNA disintegration. The doomed spermatogonia also showed very weak LysoTracker staining (Fig. 3B). Immunostaining the testes of Rab7-YFP–expressing flies with another marker of live germ cell progenitors Lamin Dm0 (Lamin) (*27*) showed its complete degradation in TUNEL-positive debris (fig. S3A). However, we observed Rab7-YFP phagosomes around live germ cell progenitors expressing Lamin, in addition to strong DAPI but not TUNEL staining, indicating that germ cells are engulfed alive before DNA and protein degradation (fig. S3B). In addition, staining with Fei Mao (FM4-64), a membrane styryl dye, confirmed that Rab7-YFP–positive vesicles fused around LysoTracker-positive germ cells, which were engulfed within single cyst cells (fig. S4, A and B). Moreover, live imaging (*n* = 3) revealed the presence of Rab7-YFP late endosomes surrounding germ cells, which progressively stained positive for LysoTracker (Fig. 3C and movie S3). Together, these results demonstrate that single cyst cells engulf live spermatogonia.

**Fig. 4.**
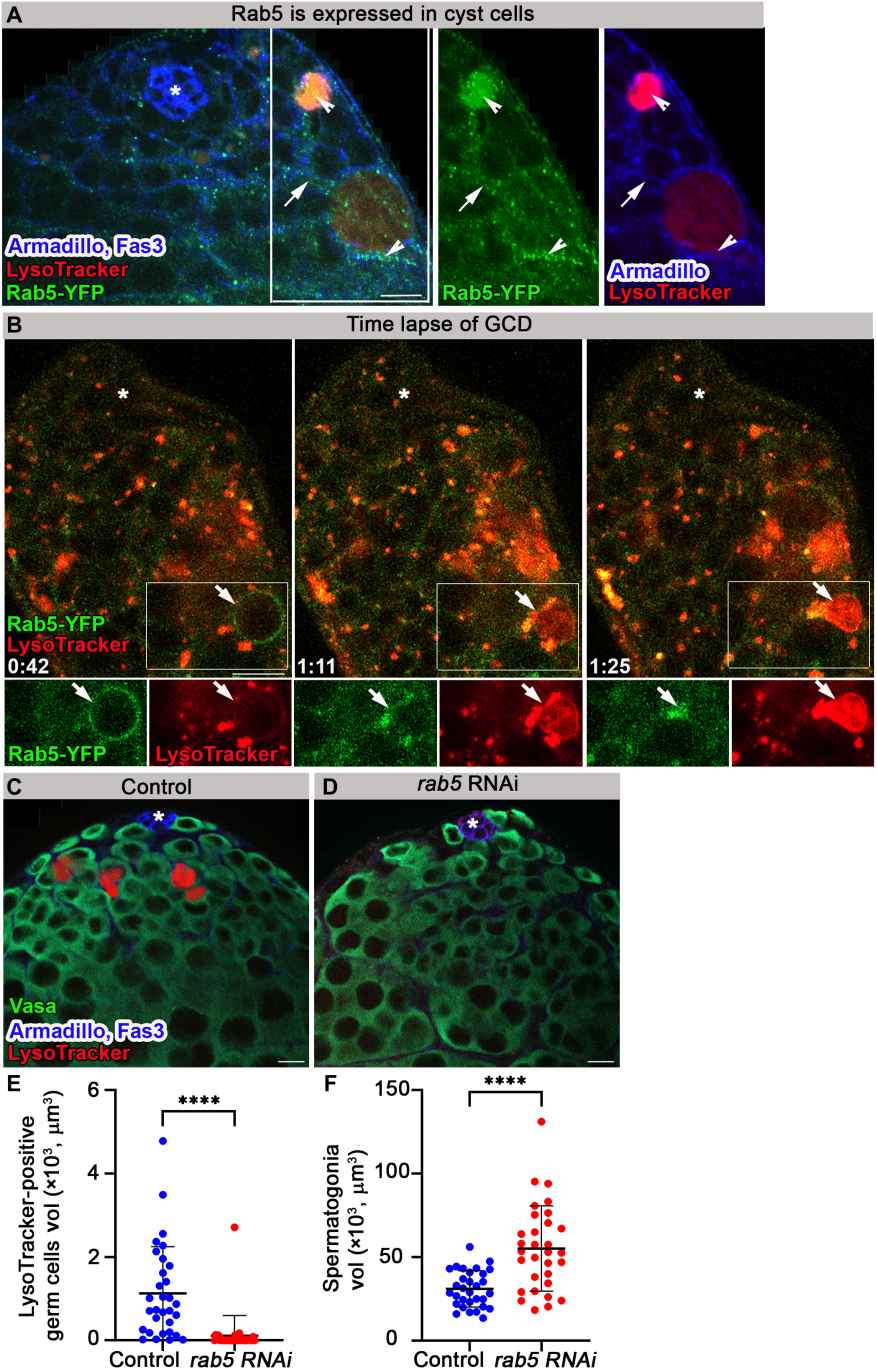
Rab5-containing early endosomes are necessary for GCD. (**A**) Immunofluorescence images of testis from flies expressing YFP at endogenous chromosomal locus of *rab5* (green, Rab5-YFP) labeled with LysoTracker (red) and Armadillo (blue, cyst and hub cells). Arrow marks Rab5 expression in cyst cells. Arrowheads mark Rab5 expression around and in dying germ cells. (**B**) Snapshots of live-imaged testis from Rab5-YFP–expressing flies (green) marked with LysoTracker (red). Insets are high-magnification views of boxed areas, highlighting early endosomes surrounding live germ cells, which are gradually filled with LysoTracker. Time (hour:min) is shown on the bottom left of the images. (**C** to **F**) Testes of 3-day control [(C) *c587Gal4;Gal80^TS^* outcrossed to *w1118*, *n* = 31] and a *rab5* RNAi transgene expressed in cyst cells of adult males by TARGET driver [(D) *c587Gal4;Gal80^TS^,UAS-rab5RNAi*, *n* = 31] were stained for Armadillo and Fas3 (blue, cyst cells and hub, respectively), Vasa (green, germ cells), and LysoTracker (red). Quantification of the volume of LysoTracker-positive germ cells (E) and live spermatogonia cells (F) as measured with Imaris (control, blue dots and *rab5* RNAi, red dots). Note significant reduction in GCD in testes of *rab5* RNAi–expressing flies. Statistical significance was determined by a Mann-Whitney test, ****P ≤ 0.0001. Asterisks mark the hub, and scale bars correspond to 10 μm.

Similarly, Rab5-YFP is expressed in cyst cells and forms vesicles around dying germ cells (Fig. 4A). Immunostaining the testes of Rab5-YFP–expressing flies with Lamin showed that Lamin was completely degraded in TUNEL-positive debris (fig. S5A). However, early endosomes were generated around live germ cells before Lamin degradation, DNA disintegration, and TUNEL signal (fig. S5B). Live imaging (*n* = 3) of Rab5-YFP revealed the generation of early endosomes around germ cells that gradually filled with LysoTracker. Although the LysoTracker signal was further enhanced, Rab5-YFP was rapidly disintegrated (~30 min), suggesting that Rab5 was consecutively replaced by Rab7 (Fig. 4B and movie S4).

To assess the contribution of cyst cell–derived endosomes to GCD, temporal and regional gene expression targeting (TARGET) system was used to induce RNAi-mediated knockdown of *rab5* specifically in the cyst cells of adult flies (*28*). *rab5* RNAi was previously shown to nonautonomously induce spermatogonia proliferation and prevent differentiation after 5 days at the restrictive temperature [29°C; (*29*)]. Therefore, we examined testes from flies raised only for 3 days at the restrictive temperature, before differentiation defects are observed. Immunofluorescence staining with Vasa and LysoTracker or TUNEL revealed that *rab5* RNAi markedly reduced GCD (Fig. 4, C to F, and fig. S5, C to E). Quantification indicated a 10-fold decrease in the volume of LysoTracker- and TUNEL-positive germ cells (Fig. 4E and fig. S5E). In addition, the volume of live spermatogonia increased by 1.8-fold (Fig. 4F), which is probably due to the nonautonomous effect of Rab5 on germ cell proliferation (*29*). These data show that Rab5 is necessary for GCD induction, proving the cell nonautonomous nature of the process.

### PS exposure follows lysosomal activity

**Fig. 5.**
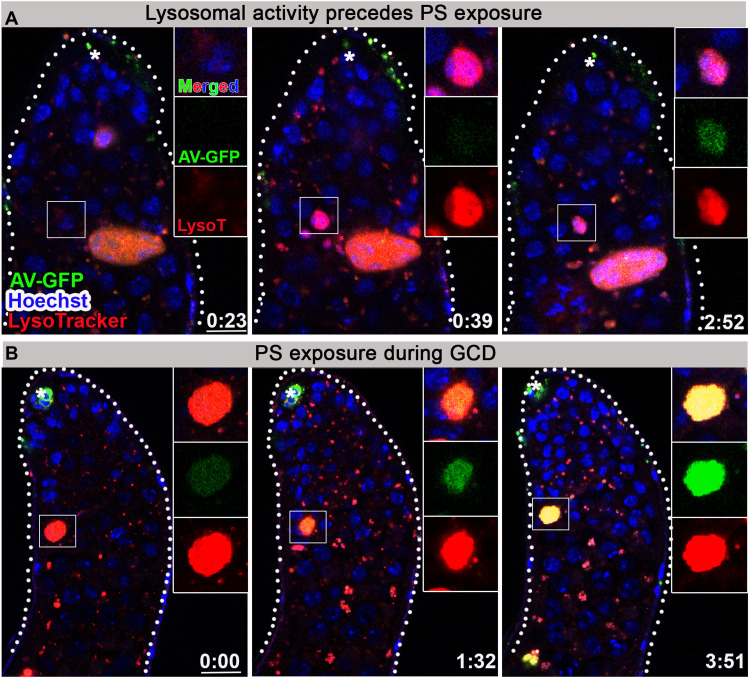
PS is exposed by dying germ cells after lysosomal activity. (**A** and **B**) Snapshots of live-imaged testes, labeled with LysoTracker (red), Hoechst (blue, nuclei), and AV-GFP expressed in and secreted from hub cells (green, *updGal4;UAS-AV-GFP*; hub cells are indicated by asterisk). Insets are high-magnification views of boxed regions highlighting progression of GCD. (**A**) Live germ cell marked only by Hoechst first becomes positive for lysosomal activity and only after ~2 hours are labeled by AV-GFP indicating PS exposure. (**B**) AV-GFP signal of PS exposure accumulates and progresses for ~4 hours. Time (hour:min) is shown on the bottom right of the images, and scale bars correspond to 10 μm.

The most common eat-me signal that leads to phagoptosis is exposure of PS on the cell surface of the live cell. To visualize the dynamics of PS externalization on germ cell membranes in vivo, the secreted PS-binding protein, annexin V (AV), was expressed as a superfolder GFP fusion protein (AV-GFP), whose fluorescence remains stable in the acidic environment of the lysosomes (*30*, *31*). This reporter successfully labeled exposed PS on apoptotic neurons of the central nervous system when expressed in embryonic macrophages, used as a positive control to confirm its secretion and diffusion within the tissue to label remote apoptotic cells in a proven cell death context (fig. S6A) (*32*). In a similar manner, AV-GFP was expressed in the apical hub cells of the testes, to enable specificity of the signal only to PS-exposing cells. The secreted AV-GFP can then diffuse from the hub cells to the extracellular space surrounding the more distant cyst and germ progenitor cells, where it can bind any exposed PS (Fig. 5, A and B, and movie S5). Using live imaging (*n* = 6), we were unable to detect AV-GFP on germ cells before lysosomal activity (Fig. 5A). However, dying germ cells already marked with LysoTracker revealed very weak AV-GFP staining, which was further enhanced and accumulated for 4 hours along with GCD progression (Fig. 5B and movie S5). In contrast, AV(mut)-GFP, which bears mutations that eliminate its ability to interact with PS (*31*, *33*) and serves as a negative control, did not label any germ cells (fig. S6, B and C, and movie S6). The observed PS marking could indicate late exposure on the outer leaflet of the germ cell membranes or gradual loss of membrane integrity. In any event, PS exposure did not mark germ cells before induction of GCD and thus cannot act as a classic eat-me signal, although we cannot rule out the possibility that PS exposure below the detection level precedes lysosomal activity.

### Cyst cell membranes penetrate into dying germ cells

**Fig. 6.**
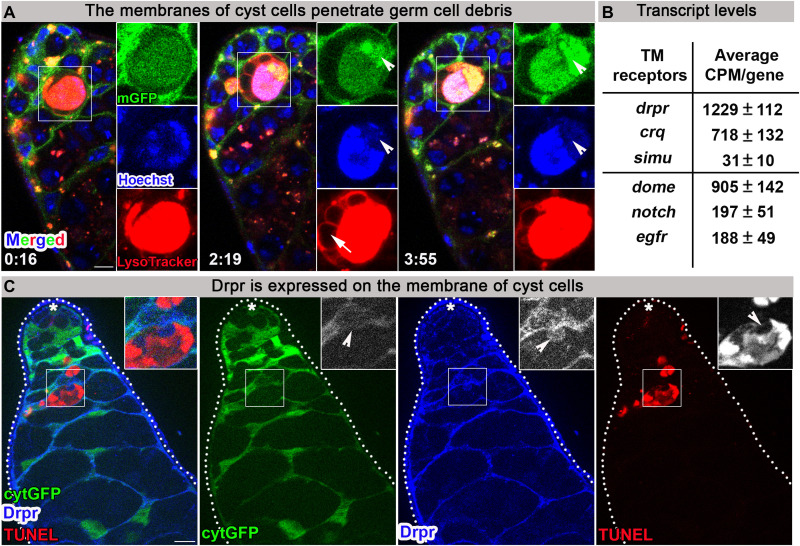
The phagocytic transmembrane receptor Drpr is expressed in cyst cells. (**A**) Snapshots of live-imaged testis, marked with LysoTracker (red), Hoechst (blue, nuclei), and a membrane-targeted mGFP (cyst cells, *c587Gal4;UAS-mGFP*). Insets are high-magnification views of boxed regions, highlighting progression of GCD. Arrowheads mark membrane accumulation in DNA degradation domain. Arrow marks LysoTracker-free blebs surrounding dying germ cell. Time (hour:min) is shown on the bottom left of the images. (**B**) mRNA levels of six transmembrane (TM) receptors analyzed from the transcriptome of cDNA libraries prepared from RNA samples (four biological repeats) extracted from testes of wild-type young males (2 days old). Shown are transcript levels of three phagocytic TM receptors [*drpr*, *simu*, and *crq* (upper)] and three TM receptors previously reported as expressed in the apical tip of the testis [*dome*, *notch*, and *egfr* (lower)]. (**C**) The apical tip of a testis that expresses cytGFP in cyst cells (green, *c587Gal4;UAS-cytGFP*), immunostained for Drpr (blue) and TUNEL (red, dying germ cells). Insets are high-magnification views of boxed regions, highlighting how the cyst cell membrane expressing Drpr protrudes into dying germ cell (arrowheads). Asterisks mark the hub, and scale bars correspond to 10 μm.

Live imaging of cyst cells marked with cytGFP revealed their dynamic morphology as they stretch significantly to engulf the large cluster of dying germ cells (see above; Fig. 1D and movie S1). To directly investigate the cyst cell membranes that mediate the interaction with germ cells, a membrane-targeted CD8-GFP (mGFP) (*34*) was used. Unexpectedly, live imaging of mGFP (*n* = 3) with LysoTracker and Hoechst revealed an active role for the cyst cell membranes in DNA degradation of dying germ cells. At early stages of GCD, when the DNA is still packed in single nuclei, cyst cell membranes interact uniformly with the entire dying spermatogonia. However, as GCD progresses and the chromatin involutes, the membranes concentrate at DNA degradation domains (Fig. 6A and movie S7). This observation suggests that cyst cell–derived transmembrane and associated proteins may be involved in germ cell phagoptosis. In most of the imaged testes, blebs were detected pinching off the dying germ cells. While the surfaces of these blebs were positive for mGFP and LysoTracker, the inner content was negative for these markers. Intriguingly, live imaging of AV-GFP secreted from hub cells revealed AV-GFP within the LysoTracker-free blebs (fig. S6D), suggesting that phospholipids of the dying germ cells are transported to the cyst cells during GCD via these vesicles, which may serve as a transport mechanism to recycle components of the dying germ cells.

### The phagocytic receptor Drpr is expressed in cyst cells

To identify those phagocytic transmembrane receptors in cyst cells that mediate the interaction with germ cells, we first consulted our previously reported transcriptome analysis of complementary DNA (cDNA) libraries generated from testes of wild-type adult males (*w1118*) (*35*). Except for *six microns under* (*simu*) (*36*) that presented low expression, mRNA levels of the established transmembrane phagocytic receptors *drpr* and *croquemort* (*crq*) (*37*) were found to be high and comparable to the levels of known transmembrane receptors expressed at the apical tip of the testis, namely, *domeless* (*dome*), *notch*, and *epidermal growth factor receptor* (*egfr*) (*38*–*41*) (Fig. 6B).

We next performed immunofluorescence analysis to determine whether any of these phagocytic receptors are expressed in cyst cells. Immunostaining with anti-SIMU antibodies did not reveal detectable expression of this protein at the apical tip of the testis. However, Drpr, which is the most highly expressed receptor in the transcriptome (Fig. 6B), was detected on the membranes of cyst cells that encapsulate spermatogonia. Moreover, these images revealed Drpr-labeled protrusions extending from cyst cells into dying germ cells (Fig. 6C). As a negative control, Drpr signal was not observed in testes of *drpr*-null mutants (*42*) (fig. S7, A and B), confirming the specificity of the anti-Drpr antibody.

### Hyperplasia of spermatogonia cells and delayed degradation are observed at the apical tip of *drpr*-null mutants

**Fig. 7.**
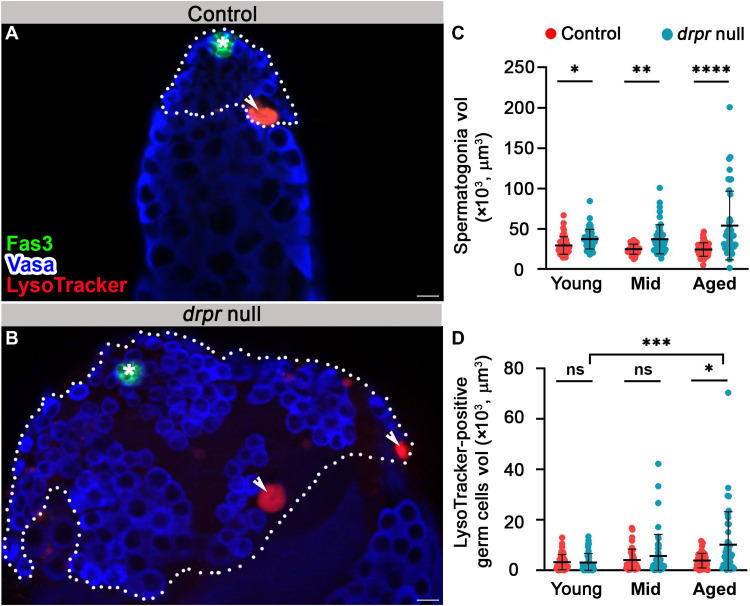
Hyperplasia of progenitor germ cells and attenuated degradation in testes of *drpr*-null males. (**A** and **B**) Representative images of the apical tip of testes from 7-day-old males of control [(A) *w1118*] and *drpr*-null flies (B). Testes were immunostained for Fas3 (green, hub), Vasa (blue, germ cells), and LysoTracker (red, dying germ cells). The white dashed line delineates spermatogonia cells. Asterisks mark the hub, arrowheads mark LysoTracker-positive germ cells, and scale bars correspond to 10 μm. (**C** and **D**) Quantification of the volume of live spermatogonia cells (C) and LysoTracker-positive germ cells (D) as measured with Imaris in control (red dots, *w1118*) and *drpr* null (blue dots). Young: control (*n* = 48) and *drpr* null (*n* = 49); mid-aged: control (*n* = 42) and *drpr* null (*n* = 48); and aged: control (*n* = 47) and *drpr* null (*n* = 41). Note the age-dependent increases in hyperplasia and volume of germ cell debris only in *drpr* null samples. Statistical significance was determined by a Kruskal-Wallis test; *P ≤ 0.05, **P ≤ 0.01, ***P ≤ 0.001, and ****P ≤ 0.0001; ns, not significant.

To determine whether Drpr, which has previously been shown to mediate engulfment (*20*), plays a role in phagoptosis of spermatogonia, testes from *drpr*-null flies (*42*) were examined following staining with Vasa to mark live germ cells and with LysoTracker to mark germ cell debris. We then compared the volume of live spermatogonia and germ cell debris in testes from young (1 to 2 days), mid-aged (7 days), and aged (30 days) males (Fig. 7, A to D). The most obvious difference between control and *drpr*-null testes, observed in all three age groups, was a significantly enlarged apical tip filled with excess spermatogonia cells (Fig. 7, A to C). There was no significant difference in the sizes of single germ cells, indicating that in *drpr*-null males, expansion occurred by hyperplasia of spermatogonia cells rather than by hypertrophy of individual cells. The hyperplasia of *drpr*-null testes can result from the accumulation of cells that divide excessively and/or escape cell death. Significantly, immunostaining with anti–phospho-Thr 3-histone H3 (pHH3) antibodies to mark mitotic spermatogonia events actually depicted less proliferation in the testis of *drpr*-null males (fig. S7, C to E), ruling out enhanced proliferation as a cause of the hyperplasia. These results indicate that the accumulation of spermatogonia results from cells that failed to undergo cell death. If Drpr solely mediates clearance of dead cells, as occurs at the last step of cell autonomous death processes such as apoptosis, then one would expect more debris to accumulate in the testes of *drpr*-null mutants compared with control in each of the aged groups. Yet, when compared with young and mid-aged controls, the mean volume of LysoTracker- or TUNEL-positive debris in the testes of *drpr*-null males did not change significantly (Fig. 7, A, B, and D, and fig. S7, F to H). The lack of accumulation of cell debris combined with the increased number of germ cells observed in the apical tip indicates that in this system, Drpr’s engulfment function contributes to the elimination of live germ cells.

In aged males, the volume of debris increased significantly in *drpr*-null testes compared with controls (Fig. 7D), in which the average volume of debris remained constant in all age groups. This suggests that debris lingers for a longer time and that the rate of degradation in the testes of *drpr*-null flies is significantly slower, which is consistent with several studies showing an additional role for Drpr in the degradation of cellular debris (*36*, *43*). Together, these results indicate that there may be fewer GCD events in testes of *drpr*-null that linger for a longer time.

**Fig. 8.**
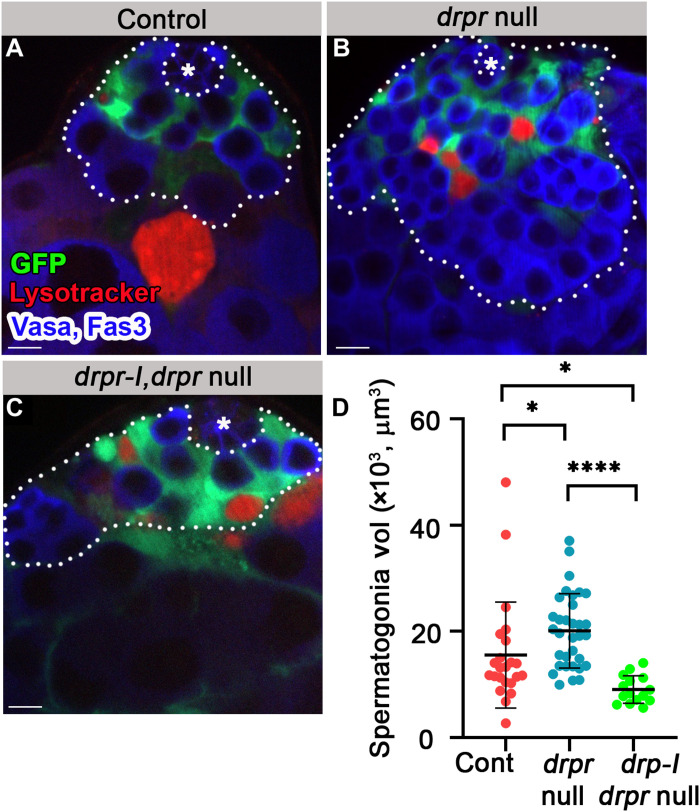
Drpr-I expression in the cyst cells of *drpr*-null flies rescues hyperplasia. (**A** to **D**) Testes from 7-day-old control [(A) *c587Gal4;UAS-cytGFP;drpr* null/TM6, *n* = 23], *drpr* null [(B) *c587Gal4;UAS-cytGFP*;*drpr* null, *n* = 33], and *drpr-I, drpr* null [(C) *c587Gal4;UAS-cytGFP,UAS-drpr-I;drpr* null, *n* = 15] males. Testes expressing GFP in cyst cells (green) immunostained for Fas3 and Vasa (blue, hub and germ cells) and LysoTracker (red, dying germ cells). The white dashed line delineates spermatogonia cells. Asterisks mark the hub, and scale bars correspond to 10 μm. (D) Quantification of the volume of spermatogonia cells as measured with Imaris. Note that *drpr-I* expression in cyst cells rescues the hyperplasia of *drpr*-null flies. Statistical significance was determined by a Kruskal-Wallis test; *P ≤ 0.05 and ****P ≤ 0.0001.

Alternative splicing of the *drpr* gene can yield six receptor isoforms, each of which consists of unique extracellular and intracellular sequences, but only one of which, Drpr-I, has the ITAM (immunoreceptor tyrosine-based activation motif) that mediates pro-phagocytic activity (*44*). To directly address the possibility that the hyperplasia phenotype of *drpr*-null is due to a lack of the pro-phagocytic Drpr-I isoform in cyst cells, mutant cyst cells were transformed to express Drpr-I (*c587Gal4;UAS-cytGFP,UAS-drpr-I;drpr* null). *drpr-I* expression rescued the accumulation of live spermatogonia (Fig. 8, A to D).

In summary, the age-related accumulation of debris volume in *drpr*-null males, together with the significantly greater number of live spermatogonia, suggests that Drpr is required within cyst cells to nonautonomously engulf live germ cells and degrade the resulting debris, further supporting the hypothesis that cyst cells phagoptose germ cells.

### Crq and Ced-12 nonautonomously promote GCD

Unlike the *rab5* and *lamp1* RNAi, however, where GCD was significantly reduced (see above; Figs. 2, F and G, and 4E and fig. S5E), GCD still occurred in *drpr*-null testes, probably because of redundant function of additional transmembrane phagocytosis receptors. Crq, the second transmembrane receptor identified in our transcriptome analysis, is a CD36-related receptor expressed in macrophages (*37*) that promotes nurse cells death in the *Drosophila* ovary (*45*). Immunofluorescence analysis of the apical tip of the testes revealed Crq expression in a punctate pattern in the cytoplasm of cyst cells (fig. S8A). The punctate expression pattern of Crq was also observed in the cytoplasm of embryonic macrophages (*46*). To determine whether Crq has a nonautonomous role in GCD induction, RNAi was used to reduce its levels in cyst cells (fig. S8B). *crq* RNAi did not affect the average volume of spermatogonia but significantly reduced the volume of LysoTracker- and TUNEL-positive germ cells, suggesting that Crq is involved in GCD (fig. S8, C to E).

To further investigate the engulfment of live germ cells, RNAi to *cell death abnormality (ced)–12* was expressed in cyst cells. While Ced-12 is not a transmembrane receptor and its upstream regulator in *Drosophila* is unknown, it activates the GTPase Rac-1 during glial engulfment (*47*) and promotes apoptotic clearance in ovarian follicle cells (*45*). *ced-12 RNAi*–expressing males showed a similar phenotype to *drpr* nulls, with a significantly increased spermatogonia volume, but without affecting the volume of LysoTracker-positive germ cells (fig. S8, F to I).

### *Drpr* acts in parallel with *ced-12* and *crq* and upstream of *lamp1* during GCD

**Fig. 9.**
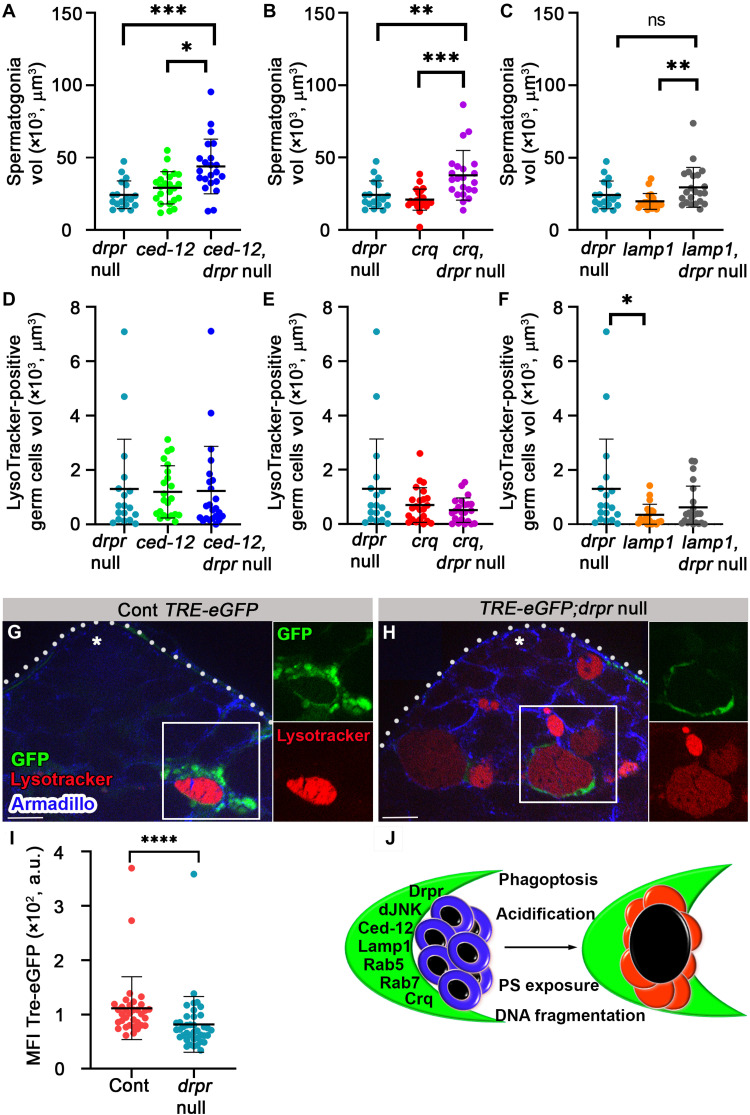
*drpr* acts in parallel with *ced12* and *crq* and upstream of *lamp1* and dJNK in cyst cells during phagoptosis. (**A** to **F**) Quantification of the volume of live spermatogonia cells (A to C) and LysoTracker-positive germ cells (D to F) as measured with Imaris in testes from *drpr* null (*c587Gal4;UAS-cytGFP/CyO;drpr* null, *n* = 18), *ced-12* RNAi (*c587-Gal4;UAS-cytGFP/UAS-ced-12 RNAi*; *drpr* null/TM2, *n* = 23), *ced-12* RNAi and *drpr*-null double mutants (*c587-Gal4;UAS-cytGFP/UAS-ced-12 RNAi*; *drpr* null, *n* = 22), *crq* RNAi (*c587-Gal4;UAS-cytGFP/UAS-crq RNAi*; *drpr* null/TM2, *n* = 23), *crq* RNAi and *drpr*-null double mutants (*c587-Gal4;UAS-cytGFP/UAS-crq RNAi*; *drpr* null, *n* = 22), *lamp1* RNAi (*c587-Gal4;UAS-cytGFP/UAS-lamp1 RNAi*; *drpr* null/TM2, *n* = 21), and *lamp1* RNAi and *drpr*-null double mutants (*c587-Gal4;UAS-cytGFP/UAS-lamp1 RNAi*; *drpr* null, *n* = 22). Note that *drpr* acts in parallel with *ced-12* and *crq* in engulfment and upstream of *lamp1*. Statistical significance was determined by a Kruskal-Wallis test; *P ≤ 0.05, **P ≤ 0.01, and ***P ≤ 0.001. (**G** and **H**) Expression of TRE-eGFP reporter in wild-type (G) and *drpr*-null testes (H) labeled with LysoTracker (red) and immunostained for Armadillo (blue, cyst cells). Insets are views of single channels of boxed regions. Asterisks mark the hub, and scale bars correspond to 10 μm. (**I**) The mean fluorescence intensity (MFI) of GFP signal quantification (*n* = 42 regions of germ cell debris in each genotype) is shown. Note the reduced GFP signal in the testis of *drpr*-null animals. Statistical significance was determined by a Mann-Whitney test, ****P ≤ 0.0001. (**J**) Schematic summarizing how cyst cells (green) induce phagoptosis of live germ cells (blue, left) resulting in lysosomal activity, DNA fragmentation, and PS exposure in germ cells (red, right).

Drpr and Ced-12 were shown to work in parallel pathways in *Drosophila* hemocytes (*48*) or in the same pathway in glia (*47*). To explore the genetic interactions between *drpr* and each of the above phagocytic factors, we generated *drpr* mutants expressing RNAi lines of *ced-12*, *crq*, or *lamp1* specifically in cyst cells and measured the volumes of live spermatogonia and LysoTracker-positive debris. Analyzing double mutants for *drp*r and *ced-12* or *drpr* and *crq* revealed additive increases in live spermatogonia, as compared with the single mutants, suggesting their independent and parallel functions in engulfment (Fig. 9, A and B). Notably, *crq* RNAi alone did not increase live spermatogonia, suggesting that Drpr is the main engulfing receptor, while Crq is likely required when Drpr is absent (figs. S8E and Fig. 9B). The double mutants did not significantly affect the volume of LysoTracker-positive debris, indicating that Ced-12 and Crq are not involved in degradation (Fig. 9, D and E). Examination of *drpr* and *lamp1* double mutants revealed similar live spermatogonia volume and LysoTracker-positive debris as with *drpr* single mutants, reflecting that *lamp1* acts downstream of *drpr* in a common pathway (Fig. 9, C and F).

### Drpr activates *Drosophila* c-Jun N-terminal kinase signaling in cyst cells

Drpr has been shown to induce *Drosophila* c-Jun N-terminal kinase (dJNK) signaling in stretch follicle cells during developmental death of ovarian nurse cells, and in the cyst cells following starvation-induced GCD (*17*, *49*). Under normal conditions, basal levels of dJNK are required to maintain cyst stem cell (CySC) residency at the apical niche, and down-regulation of dJNK reduces the number of both CySCs and cyst cells (*50*). Since we therefore could not use knockdown strategies to determine whether dJNK has a nonautonomous role in GCD induction downstream to Drpr, we used a reporter assay to assess its activation in cyst cells during spontaneous GCD. dJNK signaling is a critical mediator of phagocytic activity that stimulates the downstream target AP1, as has been shown in glial cells (*51*–*53*). *TRE-eGFP*, a reporter comprising AP1 sites fused to an eGFP sequence, was introduced to both wild-type and *drpr*-null mutant flies (*51*). In wild-type samples, the *TRE-eGFP* reporter was activated in all cyst cells, with stronger induction in cyst cells that surrounded dying germ cells (Fig. 9G and movie S8). Compared with the wild-type males, there was a significant decrease in *TRE-eGFP* reporter induction in cyst cells surrounding the debris of *drpr*-null mutants (Fig. 9, G to I). These results suggest that Drpr acts upstream of dJNK within cyst cells.

## DISCUSSION

In the present study, we describe a cell nonautonomous death process by which cyst cells kill live spermatogonia progenitors within the *Drosophila* adult testes. This function joins the more well-recognized roles of cyst cells in structural support and secretion of factors that promote spermatogonia differentiation (*15*, *54*, *55*). Detailed analysis of the sequence of events during GCD showed activation of the phagocytic machinery, i.e., generation of endosomal and lysosomal vesicles, within phagocytic cyst cells before protein degradation and detectable signals from the dying germ cells. The latter first included acidification of the germ cell indicative of lysosomal activity, then DNA involution and degradation, and, last, PS exposure. In addition, cyst cell membrane was shown to penetrate into the dying germ cells. Most significantly, components of the cyst cell phagocytic machinery, namely, Rab5, Lamp1, Ced-12, Crq, and Drpr, were required for GCD. Last, blebs that pinch off from the surface of the dying cell seem to recycle components toward the cyst cell. Together, these data prove that the underlying mechanism of GCD is phagoptosis by cyst cells (Fig. 9J).

Phagoptosis was shown previously to result from reversible exposure of eat-me signals on the cell surface of a doomed but live cell, which instruct phagocytic cells to engulf and consume the cell to death. In the case of germ cell phagoptosis shown here, the common signal of PS exposure on the cell surface was not detectable before signs of lysosomal activity, implying that in this scenario, PS does not function as the eat-me signal to induce phagoptosis by the cyst cell. Germ cell phagoptosis differs from other scenarios in that the cyst cells already encapsulate the germ cells. We thus speculate that the classic eat-me signals may not be necessary, although we cannot exclude that signals other than PS may serve the same purpose. It is not yet known whether activation of the cyst cell’s endosomal and lysosomal machinery is a random event or whether there is some signal that leads to its activation. A putative signal may activate Drpr, which functions in a dual capacity during phagoptosis, namely, engulfment of the germ cells and its subsequent degradation. Furthermore, Drpr induces JNK signaling within the cyst cells surrounding the doomed germ cell syncytia. Considering that phagoptosis is only partially suppressed in *drpr*-null mutants, additional factors are most likely involved in the process. These include Ced-12 and Crq, which also act in cyst cells to nonautonomously regulate engulfment of live germ cells and induction of GCD.

A parallel can be made between the role that cyst cells play in phagoptosis of excess sperm progenitors to that of epithelial stretch follicle cells during developmental cell death of germline nurse cells in the *Drosophila* ovary. During the latter, follicle cells stretch to surround and phagoptose the nurse cells, a process that also requires Drpr (*4*, *56*). Cyst cells have also been shown to be necessary for starvation-induced death of spermatogonia during early stages of transit amplifying division in the fly testis. Here, too, they act as phagocytes to consume progenitor debris in a nonautonomous manner, recycling cellular material to provide energy and ensure survival of GSCs (*17*). It differs, however, from the developmental GCD that we report here, in that during starvation, cyst cells undergo apoptosis, which is required for the subsequent death of the germ cells. Nevertheless, it appears that phagoptosis by ancillary cells is a recurring theme in death of the germline in *Drosophila*. This cell nonautonomous form of death may predominate in germ cell development, rather than apoptosis, since the function of caspases may have been diverted from cell death to germ cell differentiation (*57*).

Previously, GCD of the fly testes was characterized as a nonapoptotic mechanism involving lysosomal activity and acidification of germ cells (*12*). Autophagy-dependent cell death was excluded; however, lysosomal factors such as Cathepsin D, deep orange, and Carnation were shown to be involved, although it was not indicated in which cell type, i.e., the cyst or germ cells, they functioned. These data are all consistent with the phagoptosis mechanism that we propose here. In addition, mitochondria were shown to play an important role in GCD, as several mitochondrial proteins, such as HtrA2/Omi, Pink1, endonuclease G, Debcl, and Buffy, were shown to be necessary for death of spermatogonia (*12*). We do not know whether these factors serve to initiate a signal that activates the cyst cell to phagoptose the germ cell or whether this occurs in parallel to the events we describe here. More research will be required to fully reconcile the data.

Constant spontaneous GCD in the adult testis has also been reported in several mammalian species (*12*–*14*). Morphological features of germ cell debris and the phenotypes of mouse mutants demonstrated that also in mammals, the process of GCD deviates from the conventional apoptotic program (*13*, *58*). Moreover, phagocytic Sertoli cells that line the seminiferous tubules of mammalian testes display the double-edged sword function of both supporting the developing germ cells and clearing the resulting debris (*59*). Since many functional and molecular features of spermatogenesis are evolutionarily conserved, it is tempting to propose that Sertoli cells may also have the capacity to perform phagoptosis of live progenitors. The advantage of having a cell nonautonomous rather than an autonomous type of cell death lies in the ability to regulate the death rate in response to changing external cues, such as age and nutrient availability.

## MATERIALS AND METHODS

### Experimental design: Live imaging of GCD

Chambered coverslips with eight wells (Ibidi) were covered with 0.01% poly-l-lysine (Sigma-Aldrich) for 40 min. After poly-l-lysine was removed, the wells were dried in chemical hood. Whole-mount testes from adult *Drosophila* were dissected in Ringer’s solution (128 mM NaCl, 2 mM KCl, 1.8 mM CaCl_2_, 4 mM MgCl_2_, 35.5 mM sucrose, and 5 mM Hepes, pH 6.9) containing Hoechst (Thermo Fisher Scientific, 8 μM) and LysoTracker (Thermo Fisher Scientific, 1 μM) and/or FM4-64 (Life Technologies, 5 μg/ml). The testes were gently adhered to the poly-l-lysine–coated wells, and Ringer’s solution was replaced with *Drosophila* tissue culture medium: 10% fetal bovine serum (v/v), 0.5% penicillin/streptomycin in Schneider’s medium (Sigma-Aldrich) containing Hoechst (0.3 μM), and LysoTracker (0.03 μM). The pair of testes was kept together, connected to the seminal vesicles to mimic as much as possible the in vivo conditions. Damaged or injured samples were excluded from the image analysis. A single testis was imaged every 3 min for 6 to 12 hours with ~30-μm *Z* stacks (3-μm intervals). Similar conditions have been used to image the GSC niche for 12 hours (*60*). We could often detect several GCD events in the same testis sample, each in a different stage that all progressed in a similar manner. In addition, all the genotypes and florescent markers were tested both as fixed samples and in live imaging in complementary experiments and revealed similar numbers of GCD events, morphology, and marker expression.

### Fly lines and husbandry

Flies were raised at 25°C on freshly prepared standard cornmeal molasses agar medium. Young flies were selected upon hatching and dissected within the first 3 days of life. Control and experiment animals were tested at the same times. Crosses for the inducible Gal4/Gal80/UAS TARGET system (*28*) were set up and maintained at 18°C. Adults were placed in new vials and transferred to 29°C for 1 week. The maximum amount of flies was determined according to the size of the vials or bottles to prevent crowded conditions that may induce stress. In small vials (35 ml), a maximum of 20 files (males and females) were raised. The vials were flipped every 2 days thereafter. Crosses with the Gal4/UAS system were set up and maintained at 25°C.

The fly strains used in this research were *w1118*, *c587Gal4;Sco/CyO*, *UAS-Lamp1-GFP* (Bloomington Drosophila Stock Center no. 42714); *UAS-rab5 RNAi* [Vienna Drosophila Research Center (VDRC) ID no. 103945]; *UAS-lamp1 RNAi* (VDRC ID no. 7309); *UAS-croq RNAi* (VDRC ID no. 45883); *UAS-ced12 RNAi* (VDRC ID no. 107590); *Rab5-YFP*, *Rab7-YFP*, and *lamp1-mCherry* (B. Lemaitre); *UAS-AV-GFP* and *UAS-AV(mut) GFP* (C. Han) (*31*); *TRE-eGFP* (D. Bohmann); *TRE-eGFP;drpr* null; *c587Gal4;Gal80^TS^/CyO*; *c587Gal4;UAS-cytGFP*; *c587Gal4;UAS-cytGFP;drpr* null*/TM6UBX*; *Sp/CyO;drpr* null*/TM6*; *UAS-drpr-I;TM2/TM6*; *UAS-drpr-I*, and *UAS-drpr-II* (M. A. Logan). *drpr* null was originally obtained from M. Freeman (*drpr*Δ*5* (*42*)), and flies were backcrossed for several generations before use.

### Immunofluorescence microscopy, TUNEL, and LysoTracker

Whole-mount testes from adult *Drosophila* were dissected in phosphate-buffered saline (PBS) and placed on Terasaki plates in 10 μl of fix solution [2% paraformaldehyde in PLP buffer (0.075 M lysine and 0.01 M sodium phosphate buffer, pH 7.4)] for 1 hour at room temperature, rinsed, and washed twice with PBST (PBS containing 0.5% Triton X-100), followed by standard immunofluorescence staining. Primary antibodies used in this study were as follows: mouse anti-Fas3 (DSHB 7G10, 1:20), mouse anti-Drpr (DSHB 5D14-s, 1:100), mouse anti-Lamin Dm0 (DSHB ADL84.12, 1:100), and mouse anti-Armadillo antibodies (DSHB N2 7A1, 1:20), obtained from the Developmental Studies Hybridoma Bank at the University of Iowa; guinea pig anti-Drpr (*53*) (1:100); rabbit anti-Lamp1 (*23*) (1:50, a gift from A. Jenny); and rabbit anti-Crq (1:500, a gift from N. Franc). Commercially available antibodies included rabbit anti-Vasa (Santa Cruz Biotechnology, d-260, 1:100), rabbit anti-GFP (Cell Signaling Technology, D5.1, 1:75), and rabbit anti-pHH3 antibodies (Millipore, 06-570, 1:200). Secondary antibodies were obtained from Jackson ImmunoResearch Laboratories. For LysoTracker staining, the in situ fluorescent pH indicator LysoTracker Red DND-99 (Thermo Fisher Scientific, L7528; 1:100) was used. Whole-mount testes from adult *Drosophila* were dissected in PBS and placed on Terasaki plates in 10 μl of LysoTracker reagent for 10 min at room temperature. After washing twice for 10 min with PBS, standard immunofluorescence staining was performed, as described above. TUNEL labeling of the testis was carried out using the Roche in situ PCD detection kit (TMR red, 12-156-792). After the last wash of the immunofluorescence staining protocol, the samples were fixed at room temperature for 20 min and then washed with PBST. Samples were mounted in Vectashield mounting medium containing DAPI (Vector Laboratories).

### Imaging

Microscope imaging and data analyses of all images were acquired on a Nikon A1R confocal microscope or a Zeiss AxioImager M2 microscope equipped with an ApoTome2 optical sectioning device. Images were taken with an objective Plan Apochromat 40×/1.30 or 63×/oil differential interference contrast. All images in all figures are single sections, taken with a high-resolution AxioCam HRm Rev.3 FireWire microscopy camera using Zen acquisition software and processed with Adobe Photoshop CS6.

### Quantitative and statistical analysis

Spermatogonia and GC debris volumes were detected from 10 *Z* stacks (1 μm each, above and beneath the hub) as Vasa/LysoTracker/TUNEL-positive cells. Quantification of spermatogonia and GC debris volumes was performed using Imaris (Bitplane) software with an appropriate iso-surfacing threshold. We used three markers to differentiate between spermatogonia and spermatocytes. First, the nucleus of spermatocytes (observed as an empty area or “hole” in germ cells immunostained with Vasa) is crenelated and much larger compared with the round smaller nucleus of spermatogonia. Second, in contrast to spermatogonia, in spermatocytes, only a small part of the nucleus is stained for DAPI. Last, *C587-GAL4; UAS-GFP* (used in Fig. 2 and figs. S5 and S8) shows strong GFP expression in cyst cells surrounding spermatogonia compared with spermatocytes.

To determine statistical significance, Prism GraphPad version 8 software was used. First, normality and log normality were assessed using the Shapiro-Wilk test. In all experiments, there was no normal distribution, as expected from the different sizes of interconnected 2 to 16 spermatogonia (*61*). Therefore, nonparametric tests were conducted. An average of all experiments is shown, as the mean and SD ± 95% confidence interval and the number (*n*) of testes examined. *P* values were generated using two-tailed Mann-Whitney or Kruskal-Wallis tests (depending on the number of samples) to compare time points or genotypes.

## Notes

View/request a protocol for this paper from *Bio-protocol*.

## References

[1] L. Galluzzi, I. Vitale, S. A. Aaronson, J. M. Abrams, D. Adam, P. Agostinis, E. S. Alnemri, L. Altucci, I. Amelio, D. W. Andrews, M. Annicchiarico-Petruzzelli, A. V. Antonov, E. Arama, E. H. Baehrecke, N. A. Barlev, N. G. Bazan, F. Bernassola, M. J. M. Bertrand, K. Bianchi, M. V. Blagosklonny, K. Blomgren, C. Borner, P. Boya, C. Brenner, M. Campanella, E. Candi, D. Carmona-Gutierrez, F. Cecconi, F. K. M. Chan, N. S. Chandel, E. H. Cheng, J. E. Chipuk, J. A. Cidlowski, A. Ciechanover, G. M. Cohen, M. Conrad, J. R. Cubillos-Ruiz, P. E. Czabotar, V. D’Angiolella, T. M. Dawson, V. L. Dawson, V. de Laurenzi, R. de Maria, K. M. Debatin, R. J. DeBerardinis, M. Deshmukh, N. di Daniele, F. di Virgilio, V. M. Dixit, S. J. Dixon, C. S. Duckett, B. D. Dynlacht, W. S. el-Deiry, J. W. Elrod, G. M. Fimia, S. Fulda, A. J. García-Sáez, A. D. Garg, C. Garrido, E. Gavathiotis, P. Golstein, E. Gottlieb, D. R. Green, L. A. Greene, H. Gronemeyer, A. Gross, G. Hajnoczky, J. M. Hardwick, I. S. Harris, M. O. Hengartner, C. Hetz, H. Ichijo, M. Jäättelä, B. Joseph, P. J. Jost, P. P. Juin, W. J. Kaiser, M. Karin, T. Kaufmann, O. Kepp, A. Kimchi, R. N. Kitsis, D. J. Klionsky, R. A. Knight, S. Kumar, S. W. Lee, J. J. Lemasters, B. Levine, A. Linkermann, S. A. Lipton, R. A. Lockshin, C. López-Otín, S. W. Lowe, T. Luedde, E. Lugli, M. MacFarlane, F. Madeo, M. Malewicz, W. Malorni, G. Manic, J. C. Marine, S. J. Martin, J. C. Martinou, J. P. Medema, P. Mehlen, P. Meier, S. Melino, E. A. Miao, J. D. Molkentin, U. M. Moll, C. Muñoz-Pinedo, S. Nagata, G. Nuñez, A. Oberst, M. Oren, M. Overholtzer, M. Pagano, T. Panaretakis, M. Pasparakis, J. M. Penninger, D. M. Pereira, S. Pervaiz, M. E. Peter, M. Piacentini, P. Pinton, J. H. M. Prehn, H. Puthalakath, G. A. Rabinovich, M. Rehm, R. Rizzuto, C. M. P. Rodrigues, D. C. Rubinsztein, T. Rudel, K. M. Ryan, E. Sayan, L. Scorrano, F. Shao, Y. Shi, J. Silke, H. U. Simon, A. Sistigu, B. R. Stockwell, A. Strasser, G. Szabadkai, S. W. G. Tait, D. Tang, N. Tavernarakis, A. Thorburn, Y. Tsujimoto, B. Turk, T. vanden Berghe, P. Vandenabeele, M. G. Vander Heiden, A. Villunger, H. W. Virgin, K. H. Vousden, D. Vucic, E. F. Wagner, H. Walczak, D. Wallach, Y. Wang, J. A. Wells, W. Wood, J. Yuan, Z. Zakeri, B. Zhivotovsky, L. Zitvogel, G. Melino, G. Kroemer, Molecular mechanisms of cell death: Recommendations of the nomenclature committee on cell death 2018. Cell Death Differ. 25, 486–541 (2018). 29362479 10.1038/s41418-017-0012-4 PMC5864239

[2] J. Shklover, F. Levy-Adam, E. Kurant, Apoptotic cell clearance in development. Curr. Top. Dev. Biol. 114, 297–334 (2015). 26431572 10.1016/bs.ctdb.2015.07.024

[3] M. R. Elliott, K. S. Ravichandran, The dynamics of apoptotic cell clearance. Dev. Cell 38, 147–160 (2016). 27459067 10.1016/j.devcel.2016.06.029 PMC4966906

[4] G. C. Brown, A. Vilalta, M. Fricker, Phagoptosis - Cell death by phagocytosis – Plays central roles in physiology, host defense and pathology. Curr. Mol. Med. 15, 842–851 (2015). 26511705 10.2174/156652401509151105130628

[5] G. C. Brown, J. J. Neher, Eaten alive! Cell death by primary phagocytosis: ‘Phagoptosis’. Trends Biochem. Sci. 37, 325–332 (2012). 22682109 10.1016/j.tibs.2012.05.002

[6] M. Olsson, P. A. Oldenborg, CD47 on experimentally senescent murine RBCs inhibits phagocytosis following Fcγ receptor-mediated but not scavenger receptor-mediated recognition by macrophages. Blood 112, 4259–4267 (2008). 18779391 10.1182/blood-2008-03-143008

[7] S. Jitkaew, E. Witasp, S. Zhang, V. E. Kagan, B. Fadeel, Induction of caspase- and reactive oxygen species-independent phosphatidylserine externalization in primary human neutrophils: Role in macrophage recognition and engulfment. J. Leukoc. Biol. 85, 427–437 (2009). 19106181 10.1189/jlb.0408232 PMC2653945

[8] S. M. Kelley, K. S. Ravichandran, Putting the brakes on phagocytosis: “Don’t-eat-me” signaling in physiology and disease. EMBO Rep. 22, e52564 (2021). 34041845 10.15252/embr.202152564 PMC8183410

[9] K. Hakim-Mishnaevski, N. Flint-Brodsly, B. Shklyar, F. Levy-Adam, E. Kurant, Glial phagocytic receptors promote neuronal loss in adult Drosophila brain. Cell Rep. 29, 1438–1448.e3 (2019). 31693886 10.1016/j.celrep.2019.09.086

[10] J. I. Etchegaray, A. K. Timmons, A. P. Klein, T. L. Pritchett, E. Welch, T. L. Meehan, C. Li, K. McCall, Draper acts through the JNK pathway to control synchronous engulfment of dying germline cells by follicular epithelial cells. Development 139, 4029–4039 (2012). 22992958 10.1242/dev.082776 PMC3472587

[11] F. Napoletano, B. Gibert, K. Yacobi-Sharon, S. Vincent, C. Favrot, P. Mehlen, V. Girard, M. Teil, G. Chatelain, L. Walter, E. Arama, B. Mollereau, p53-dependent programmed necrosis controls germ cell homeostasis during spermatogenesis. PLOS Genet. 13, e1007024 (2017). 28945745 10.1371/journal.pgen.1007024 PMC5629030

[12] K. Yacobi-Sharon, Y. Namdar, E. Arama, Alternative germ cell death pathway in Drosophila involves HtrA2/Omi, lysosomes, and a caspase-9 counterpart. Dev. Cell 25, 29–42 (2013). 23523076 10.1016/j.devcel.2013.02.002

[13] D. J. Allan, B. V. Harmon, S. A. Roberts, Spermatogonial apoptosis has three morphologically recognizable phases and shows no circadian rhythm during normal spermatogenesis in the rat. Cell Prolif. 25, 241–250 (1992). 1596537 10.1111/j.1365-2184.1992.tb01399.x

[14] I. Rodriguez, C. Ody, K. Araki, I. Garcia, P. Vassalli, An early and massive wave of germinal cell apoptosis is required for the development of functional spermatogenesis. EMBO J. 16, 2262–2270 (1997). 9171341 10.1093/emboj/16.9.2262 PMC1169828

[15] J. G. Lim, M. T. Fuller, Somatic cell lineage is required for differentiation and not maintenance of germline stem cells in Drosophila testes. Proc. Natl. Acad. Sci. U.S.A. 109, 18477–18481 (2012). 23091022 10.1073/pnas.1215516109 PMC3494938

[16] K. L. Lu, Y. M. Yamashita, Germ cell connectivity enhances cell death in response to DNA damage in the Drosophila testis. eLife 6, e27960 (2017). 28809158 10.7554/eLife.27960 PMC5577909

[17] A. C. Chiang, H. Yang, Y. M. Yamashita, Spict, a cyst cell-specific gene, regulates starvation-induced spermatogonial cell death in the Drosophila testis. Sci. Rep. 7, 40245 (2017). 28071722 10.1038/srep40245 PMC5223112

[18] H. Yang, Y. M. Yamashita, The regulated elimination of transit-amplifying cells preserves tissue homeostasis during protein starvation in Drosophila testis. Development 142, 1756–1766 (2015). 25968311 10.1242/dev.122663 PMC4440929

[19] J. M. MacDonald, M. G. Beach, E. Porpiglia, A. E. Sheehan, R. J. Watts, M. R. Freeman, The Drosophila cell corpse engulfment receptor Draper mediates glial clearance of severed axons. Neuron 50, 869–881 (2006). 16772169 10.1016/j.neuron.2006.04.028

[20] H. H. Wu, E. Bellmunt, J. L. Scheib, V. Venegas, C. Burkert, L. F. Reichardt, Z. Zhou, I. Fariñas, B. D. Carter, Glial precursors clear sensory neuron corpses during development via Jedi-1, an engulfment receptor. Nat. Neurosci. 12, 1534–1541 (2009). 19915564 10.1038/nn.2446 PMC2834222

[21] J. L. Scheib, C. S. Sullivan, B. D. Carter, Jedi-1 and MEGF10 signal engulfment of apoptotic neurons through the tyrosine kinase Syk. J. Neurosci. 32, 13022–13031 (2012). 22993420 10.1523/JNEUROSCI.6350-11.2012 PMC3464495

[22] S. Pulipparacharuvil, M. A. Akbar, S. Ray, E. A. Sevrioukov, A. S. Haberman, J. Rohrer, H. Krämer, Drosophila Vps16A is required for trafficking to lysosomes and biogenesis of pigment granules. J. Cell Sci. 118, 3663–3673 (2005). 16046475 10.1242/jcs.02502

[23] N. Chaudhry, M. Sica, S. Surabhi, D. S. Hernandez, A. Mesquita, A. Selimovic, A. Riaz, L. Lescat, H. Bai, G. C. MacIntosh, A. Jenny, Lamp1 mediates lipid transport, but is dispensable for autophagy in Drosophila. Autophagy , 1–16 (2022). 10.1080/15548627.2022.2038999 PMC9542896 35266854

[24] B. Petrignani, S. Rommelaere, K. Hakim-Mishnaevski, F. Masson, E. Ramond, R. Hilu-Dadia, M. Poidevin, S. Kondo, E. Kurant, B. Lemaitre, A secreted factor NimrodB4 promotes the elimination of apoptotic corpses by phagocytes in Drosophila. EMBO Rep. 22, e52262 (2021). 34370384 10.15252/embr.202052262 PMC8419693

[25] G. D. Fairn, S. Grinstein, How nascent phagosomes mature to become phagolysosomes. Trends Immunol. 33, 397–405 (2012). 22560866 10.1016/j.it.2012.03.003

[26] S. Dunst, T. Kazimiers, F. von Zadow, H. Jambor, A. Sagner, B. Brankatschk, A. Mahmoud, S. Spannl, P. Tomancak, S. Eaton, M. Brankatschk, Endogenously tagged rab proteins: A resource to study membrane trafficking in Drosophila. Dev. Cell 33, 351–365 (2015). 25942626 10.1016/j.devcel.2015.03.022 PMC4431667

[27] L. J. Barton, T. Duan, W. Ke, A. Luttinger, K. E. Lovander, A. A. Soshnev, P. K. Geyer, Nuclear lamina dysfunction triggers a germline stem cell checkpoint. Nat. Commun. 9, 3960 (2018). 30262885 10.1038/s41467-018-06277-z PMC6160405

[28] S. E. McGuire, Z. Mao, R. L. Davis, Spatiotemporal gene expression targeting with the TARGET and gene-switch systems in Drosophila. Sci. STKE 2004, pl6 (2004). 14970377 10.1126/stke.2202004pl6

[29] Y. Tang, Q. Geng, D. Chen, S. Zhao, X. Liu, Z. Wang, Germline proliferation is regulated by somatic endocytic genes via JNK and BMP signaling in Drosophila. Genetics 206, 189–197 (2017). 28315838 10.1534/genetics.116.196535 PMC5419469

[30] G. Koopman, C. P. Reutelingsperger, G. A. Kuijten, R. M. Keehnen, S. T. Pals, M. van Oers, Annexin V for flow cytometric detection of phosphatidylserine expression on B cells undergoing apoptosis. Blood 84, 1415–1420 (1994). 8068938

[31] M. L. Sapar, H. Ji, B. Wang, A. R. Poe, K. Dubey, X. Ren, J. Q. Ni, C. Han, Phosphatidylserine externalization results from and causes neurite degeneration in Drosophila. Cell Rep. 24, 2273–2286 (2018). 30157423 10.1016/j.celrep.2018.07.095 PMC6174084

[32] B. Shklyar, F. Levy-Adam, K. Mishnaevski, E. Kurant, Caspase activity is required for engulfment of apoptotic cells. Mol. Cell. Biol. 33, 3191–3201 (2013). 23754750 10.1128/MCB.00233-13 PMC3753910

[33] T. Dubois, J. P. Mira, D. Feliers, E. Solito, F. Russo-Marie, J. P. Oudinet, Annexin V inhibits protein kinase C activity via a mechanism of phospholipid sequestration. Biochem. J. 330, 1277–1282 (1998). 9494097 10.1042/bj3301277 PMC1219273

[34] T. Lee, L. Luo, Mosaic analysis with a repressible cell marker for studies of gene function in neuronal morphogenesis. Neuron 22, 451–461 (1999). 10197526 10.1016/s0896-6273(00)80701-1

[35] Y. Epstein, N. Perry, M. Volin, M. Zohar-Fux, R. Braun, L. Porat-Kuperstein, H. Toledano, miR-9a modulates maintenance and ageing of Drosophila germline stem cells by limiting N-cadherin expression. Nat. Commun. 8, 600 (2017). 28928361 10.1038/s41467-017-00485-9 PMC5605507

[36] E. Kurant, S. Axelrod, D. Leaman, U. Gaul, Six-microns-under acts upstream of Draper in the glial phagocytosis of apoptotic neurons. Cell 133, 498–509 (2008). 18455990 10.1016/j.cell.2008.02.052 PMC2730188

[37] N. C. Franc, J. L. Dimarcq, M. Lagueux, J. Hoffmann, R. A. Ezekowitz, Croquemort, a novel Drosophila hemocyte/macrophage receptor that recognizes apoptotic cells. Immunity 4, 431–443 (1996). 8630729 10.1016/s1074-7613(00)80410-0

[38] A. A. Kiger, D. L. Jones, C. Schulz, M. B. Rogers, M. T. Fuller, Stem cell self-renewal specified by JAK-STAT activation in response to a support cell cue. Science 294, 2542–2545 (2001). 11752574 10.1126/science.1066707

[39] A. A. Kiger, H. White-Cooper, M. T. Fuller, Somatic support cells restrict germline stem cell self-renewal and promote differentiation. Nature 407, 750–754 (2000). 11048722 10.1038/35037606

[40] C. L. Ng, Y. Qian, C. Schulz, Notch and Delta are required for survival of the germline stem cell lineage in testes of Drosophila melanogaster. PLOS ONE 14, e0222471 (2019). 31513679 10.1371/journal.pone.0222471 PMC6742463

[41] N. Tulina, E. Matunis, Control of stem cell self-renewal in Drosophila spermatogenesis by JAK-STAT signaling. Science 294, 2546–2549 (2001). 11752575 10.1126/science.1066700

[42] M. R. Freeman, J. Delrow, J. Kim, E. Johnson, C. Q. Doe, Unwrapping glial biology: Gcm target genes regulating glial development, diversification, and function. Neuron 38, 567–580 (2003). 12765609 10.1016/s0896-6273(03)00289-7

[43] J. I. Etchegaray, E. J. Elguero, J. A. Tran, V. Sinatra, M. B. Feany, K. McCall, Defective phagocytic corpse processing results in neurodegeneration and can be rescued by TORC1 activation. J. Neurosci. 36, 3170–3183 (2016). 26985028 10.1523/JNEUROSCI.1912-15.2016 PMC4792933

[44] M. A. Logan, R. Hackett, J. Doherty, A. Sheehan, S. D. Speese, M. R. Freeman, Negative regulation of glial engulfment activity by Draper terminates glial responses to axon injury. Nat. Neurosci. 15, 722–730 (2012). 22426252 10.1038/nn.3066 PMC3337949

[45] T. L. Meehan, T. F. Joudi, A. K. Timmons, J. D. Taylor, C. S. Habib, J. S. Peterson, S. Emmanuel, N. C. Franc, K. McCall, Components of the engulfment machinery have distinct roles in corpse processing. PLOS ONE 11, e0158217 (2016). 27347682 10.1371/journal.pone.0158217 PMC4922577

[46] E. Shlyakhover, B. Shklyar, K. Hakim-Mishnaevski, F. Levy-Adam, E. Kurant, Drosophila GATA factor serpent establishes phagocytic ability of embryonic macrophages. Front. Immunol. 9, 266 (2018). 29568295 10.3389/fimmu.2018.00266 PMC5852079

[47] T. Y. Lu, J. Doherty, M. R. Freeman, DRK/DOS/SOS converge with Crk/Mbc/dCed-12 to activate Rac1 during glial engulfment of axonal debris. Proc. Natl. Acad. Sci. U.S.A. 111, 12544–12549 (2014). 25099352 10.1073/pnas.1403450111 PMC4151738

[48] E. Van Goethem, E. A. Silva, H. Xiao, N. C. Franc, The Drosophila TRPP cation channel, PKD2 and Dmel/Ced-12 act in genetically distinct pathways during apoptotic cell clearance. PLOS ONE 7, e31488 (2012). 22347485 10.1371/journal.pone.0031488 PMC3275576

[49] D. P. V. Lebo, K. McCall, Murder on the ovarian express: A tale of non-autonomous cell death in the drosophila ovary. Cell 10, (2021). 10.3390/cells10061454 PMC8228772 34200604

[50] S. C. Herrera, E. A. Bach, The emerging roles of JNK signaling in Drosophila stem cell homeostasis. Int. J. Mol. Sci. 22, (2021). 10.3390/ijms22115519 PMC8197226 34073743

[51] J. M. Macdonald, J. Doherty, R. Hackett, M. R. Freeman, The c-Jun kinase signaling cascade promotes glial engulfment activity through activation of draper and phagocytic function. Cell Death Differ. 20, 1140–1148 (2013). 23618811 10.1038/cdd.2013.30 PMC3741495

[52] R. Hilu-Dadia, K. Hakim-Mishnaevski, F. Levy-Adam, E. Kurant, Draper-mediated JNK signaling is required for glial phagocytosis of apoptotic neurons during Drosophila metamorphosis. Glia 66, 1520–1532 (2018). 29520845 10.1002/glia.23322

[53] J. Shklover, K. Mishnaevski, F. Levy-Adam, E. Kurant, JNK pathway activation is able to synchronize neuronal death and glial phagocytosis in Drosophila. Cell Death Dis. 6, e1649 (2015). 25695602 10.1038/cddis.2015.27 PMC4669801

[54] M. J. Fairchild, F. Islam, G. Tanentzapf, Identification of genetic networks that act in the somatic cells of the testis to mediate the developmental program of spermatogenesis. PLOS Genet. 13, e1007026 (2017). 28957323 10.1371/journal.pgen.1007026 PMC5634645

[55] S. E. Brantley, M. T. Fuller, Somatic support cells regulate germ cell survival through the Baz/aPKC/Par6 complex. Development 146, dev169342 (2019). 30918053 10.1242/dev.169342 PMC6503986

[56] A. A. Mondragon, A. Yalonetskaya, A. J. Ortega, Y. Zhang, O. Naranjo, J. Elguero, W.-S. Chung, K. M. Call, Lysosomal machinery drives extracellular acidification to direct non-apoptotic cell death. Cell Rep. 27, 11–19.e3 (2019). 30943394 10.1016/j.celrep.2019.03.034 PMC6613820

[57] E. Arama, J. Agapite, H. Steller, Caspase activity and a specific cytochrome C are required for sperm differentiation in Drosophila. Dev. Cell 4, 687–697 (2003). 12737804 10.1016/s1534-5807(03)00120-5

[58] C. M. Knudson, K. S. Tung, W. G. Tourtellotte, G. A. Brown, S. J. Korsmeyer, Bax-deficient mice with lymphoid hyperplasia and male germ cell death. Science 270, 96–99 (1995). 7569956 10.1126/science.270.5233.96

[59] M. R. Elliott, S. Zheng, D. Park, R. I. Woodson, M. A. Reardon, I. J. Juncadella, J. M. Kinchen, J. Zhang, J. J. Lysiak, K. S. Ravichandran, Unexpected requirement for ELMO1 in clearance of apoptotic germ cells in vivo. Nature 467, 333–337 (2010). 20844538 10.1038/nature09356 PMC3773546

[60] L. J. Greenspan, E. L. Matunis, Live imaging of the Drosophila testis stem cell niche. Methods Mol. Biol. 1463, 63–74 (2017). 27734347 10.1007/978-1-4939-4017-2_4

[61] M. L. Insco, A. Leon, C. H. Tam, D. M. McKearin, M. T. Fuller, Accumulation of a differentiation regulator specifies transit amplifying division number in an adult stem cell lineage. Proc. Natl. Acad. Sci. U.S.A. 106, 22311–22316 (2009). 20018708 10.1073/pnas.0912454106 PMC2799733

