# Supplementary material for: The phagocytic cyst cells in Drosophila testis eliminate germ cell progenitors via phagoptosis
Source: Sci Adv. 2022 Jun 17;8(24):eabm4937. doi: 10.1126/sciadv.abm4937 (PMC9205596; doi:10.1126/sciadv.abm4937)
Supplement: Supplementary file 1 — Figs. S1 to S8 [file sciadv.abm4937_sm.pdf]

Science Advances
AAAS

Supplementary Materials for

The phagocytic cyst cells in *Drosophila* testis eliminate germ cell progenitors via phagoptosis

Maayan Zohar-Fux *et al.*

Corresponding author: Hila Toledano, hila@sci.haifa.ac.il

Sci. Adv. **8**, eabm4937 (2022)
DOI: 10.1126/sciadv.abm4937

The PDF file includes:

Figs. S1 to S8
Legends for movies S1 to S8

Other Supplementary Material for this manuscript includes the following:

Movies S1 to S8

Supplementary Materials

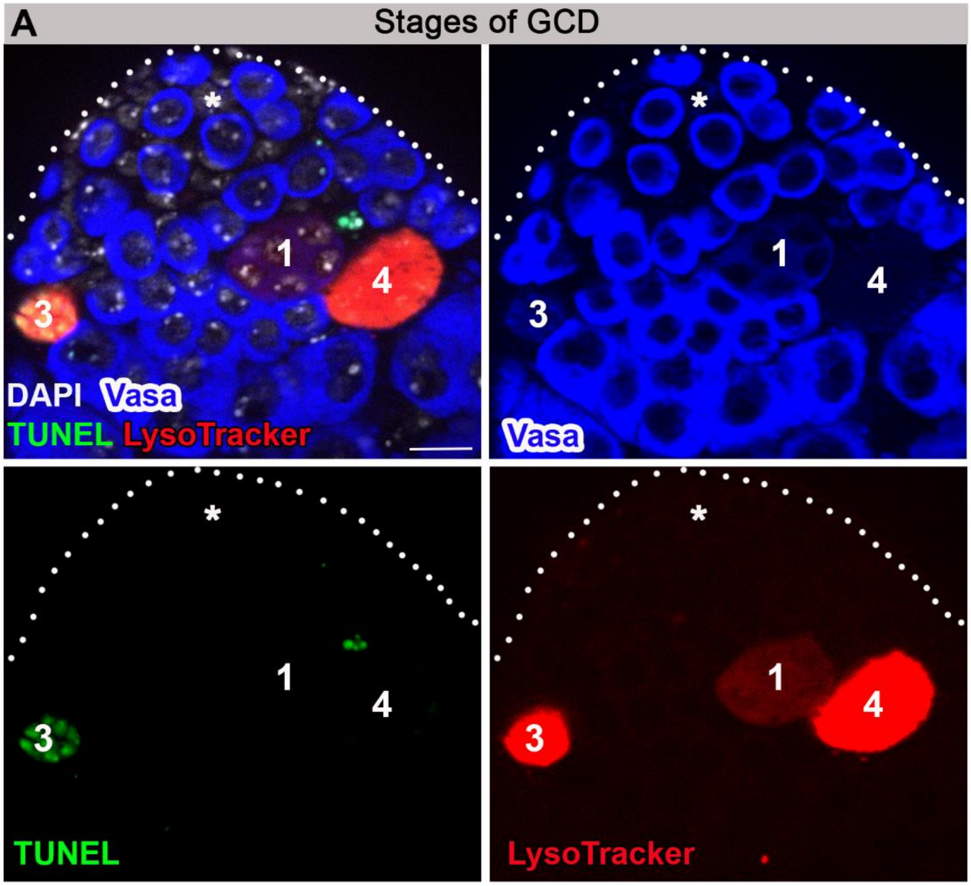

Fig. S1: Acidification precedes loss of Vasa. (**A**) Representative wild type testis (*w1118*, 2 day-old, n=49) immunostained for Vasa (blue, germ cells), TUNEL (green, fragmented DNA), LysoTracker (red, lysosomal activity) and DAPI (white, DNA). Different combination of the markers appears in stages 1, 3 and 4 of GCD progression. Note lysosomal activity at stage 1 before TUNEL appearance and Vasa loss. Asterisks mark the hub and scale bars correspond to 10µm.

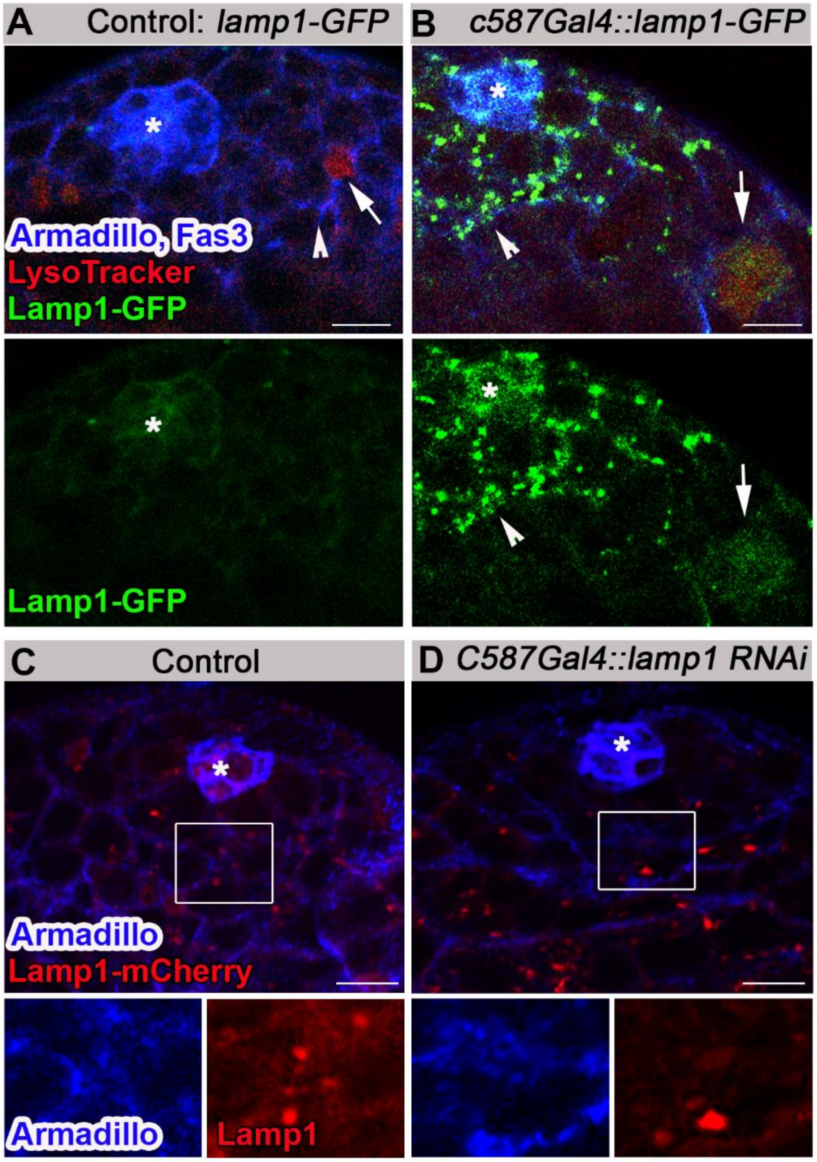

Fig. S2: Lamp1 overexpression and knockdown in cyst cells. (**A** to **B**) Immunofluorescent images of testis from control (*UAS-lamp1-GFP* alone, A) or from flies expressing Lamp1-GFP in cyst cells (green, *c587Gal4;UAS-lamp1-GFP*, B) labelled LysoTracker (red) and with Armadillo and Fas3 (blue, cyst and hub cells, respectively). Arrows mark LysoTracker positive germ cells and arrowheads mark cyst cells. Note Lamp1 punctate expression in cyst cells and no signal in control testis. **(C** to **D)** Immunofluorescent images of testes from control flies expressing mCherry at the endogenous chromosomal locus of lamp1 (C, red, *c587Gal4;lamp1-mCherry/CyO*) and a *lamp1* RNAi transgene expressed in cyst cells (D, red, *c587Gal4;lamp1-mCherry/UAS-lamp1 RNAi*) stained for Armadillo and Fas3 (blue, cyst and hub cells respectively). Bottom images are

high magnification views of the boxed regions, highlighting Lamp1-mCherry expression in cyst cells. Asterisks mark the hub and scale bars correspond to 10μm.

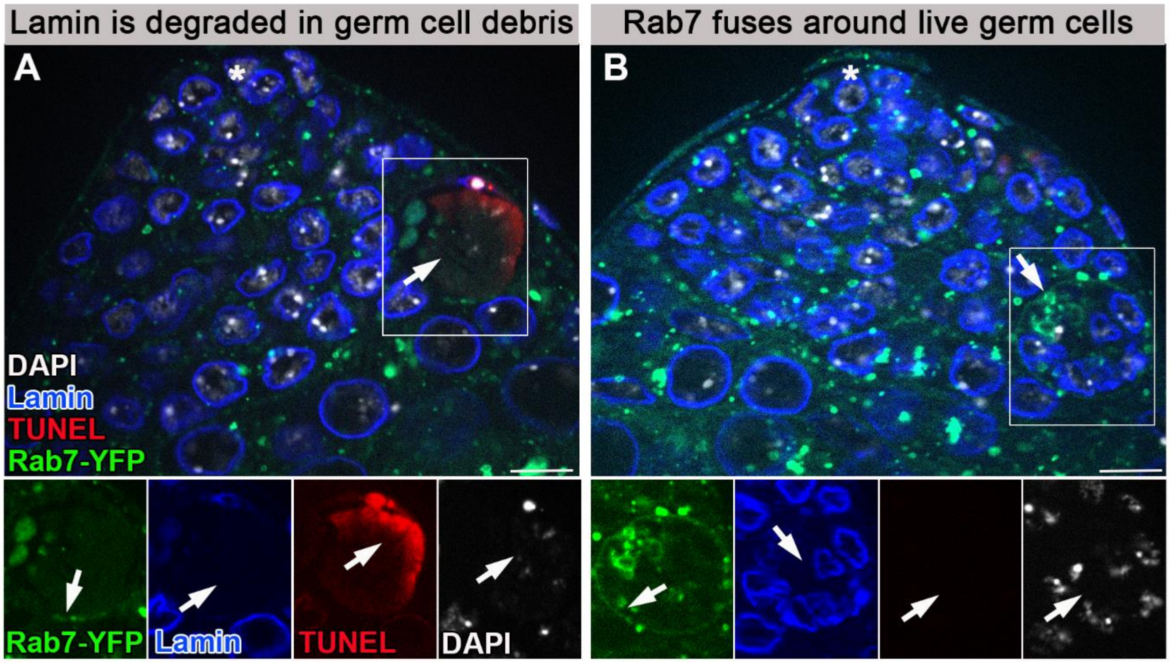

Fig. S3: Rab7 late endosomes form around live germ cells. (**A** to **B**) Immunofluorescent images of testes from flies expressing YFP at endogenous chromosomal locus of *rab7* (green, Rab7-YFP) labelled with TUNEL (red), Lamin (blue, germ cells) and DAPI (white). Bottom images are single channel views of the boxed regions. (A) Arrows highlighting Lamin and DNA degradation in TUNEL positive debris. (B) Arrows mark late endosomes surrounding live germ cells with strong Lamin and DAPI and without TUNEL signal. Asterisks mark the hub and scale bars correspond to 10μm.

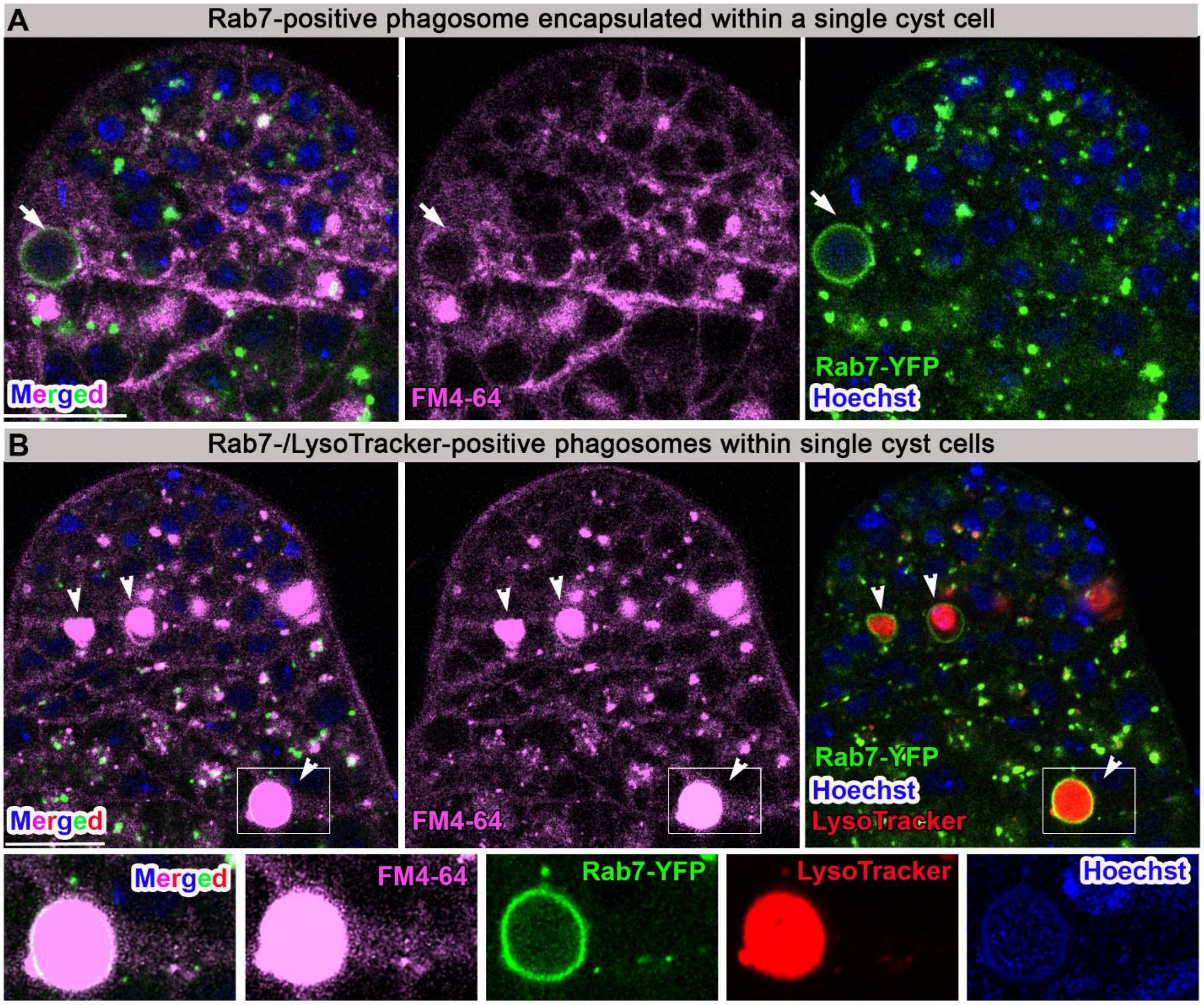

Fig. S4: Spermatogonia encapsulation by cyst cell-derived late endosomes. (**A** to **B**) Representative examples of live Rab7-YFP (green) testis stained for Hoechst (blue, nuclei) and FM4-64 (pink, cyst cell plasma membrane) without (A) or with LysoTracker (B). Note that the wide emission spectrum of LysoTracker (560-750nm) is observed also in Far Red channel used for FM4-64 detection (662.5-737.5 nm, (B)). Arrow (A) a marks Rab7-positive vesicle within a single cyst cell. Arrowheads (B) mark three GCD events and bottom images are high magnification views of one GCD event (boxed region), highlighting Rab7-positive vesicle entirely encapsulated within a single cyst cell.

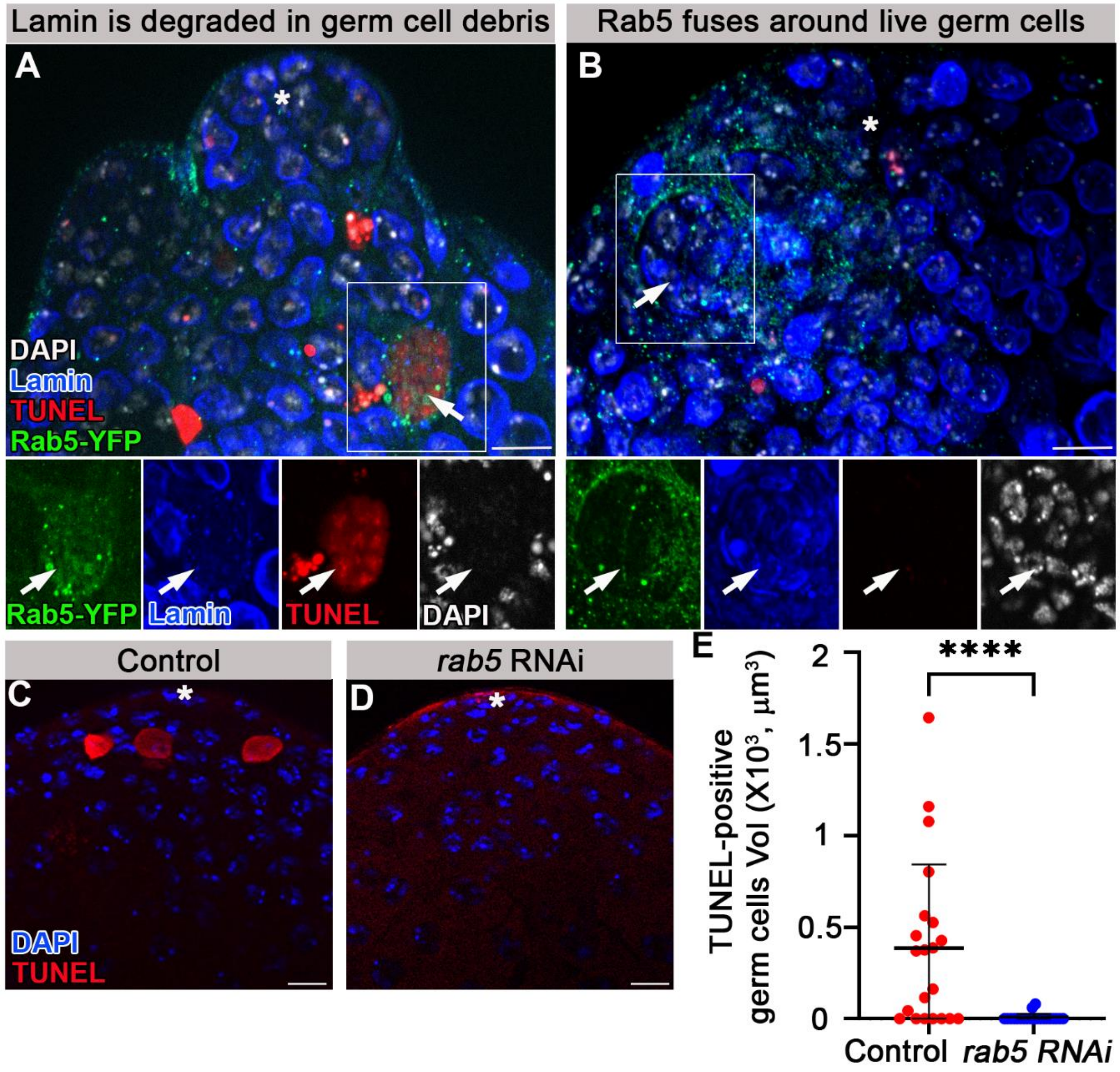

Fig. S5: Rab5 containing early endosomes fuse around live germ cells and *rab5* knockdown blocks GCD. (**A** to **B**) Immunofluorescent images of testes from flies expressing YFP at endogenous chromosomal locus of *rab5* (green, Rab5-YFP) labelled with TUNEL (red), Lamin (blue, germ cells) and DAPI (white). Bottom images are single channel views of the boxed regions. (A) Arrows highlighting Lamin and DNA degradation in TUNEL positive debris. (B) Arrows mark early endosome fusion surrounding live germ cells with strong Lamin and DAPI and without TUNEL signal. (**C** to **E**) Testes of 3-days control (C, *c587Gal4;Gal80^{TS}* outcrossed to *w1118*, n=21) and *rab5* RNAi expressed in cyst cells of adult males by TARGET driver (D, *c587Gal4;Gal80^{TS},UAS-rab5RNAi*, n=21) were stained for TUNEL (red) and DAPI (blue). (E) Quantification of the volume of TUNEL positive germ cells as measured with Imaris (control, red

dots and *rab5* RNAi, blue dots). Note significant reduction in GCD in testes of *rab5* RNAi expressing flies. Statistical significance was determined by a Mann-Whitney test, ****$p \leq 0.0001$. Asterisks mark the hub and scale bars correspond to 10μm.

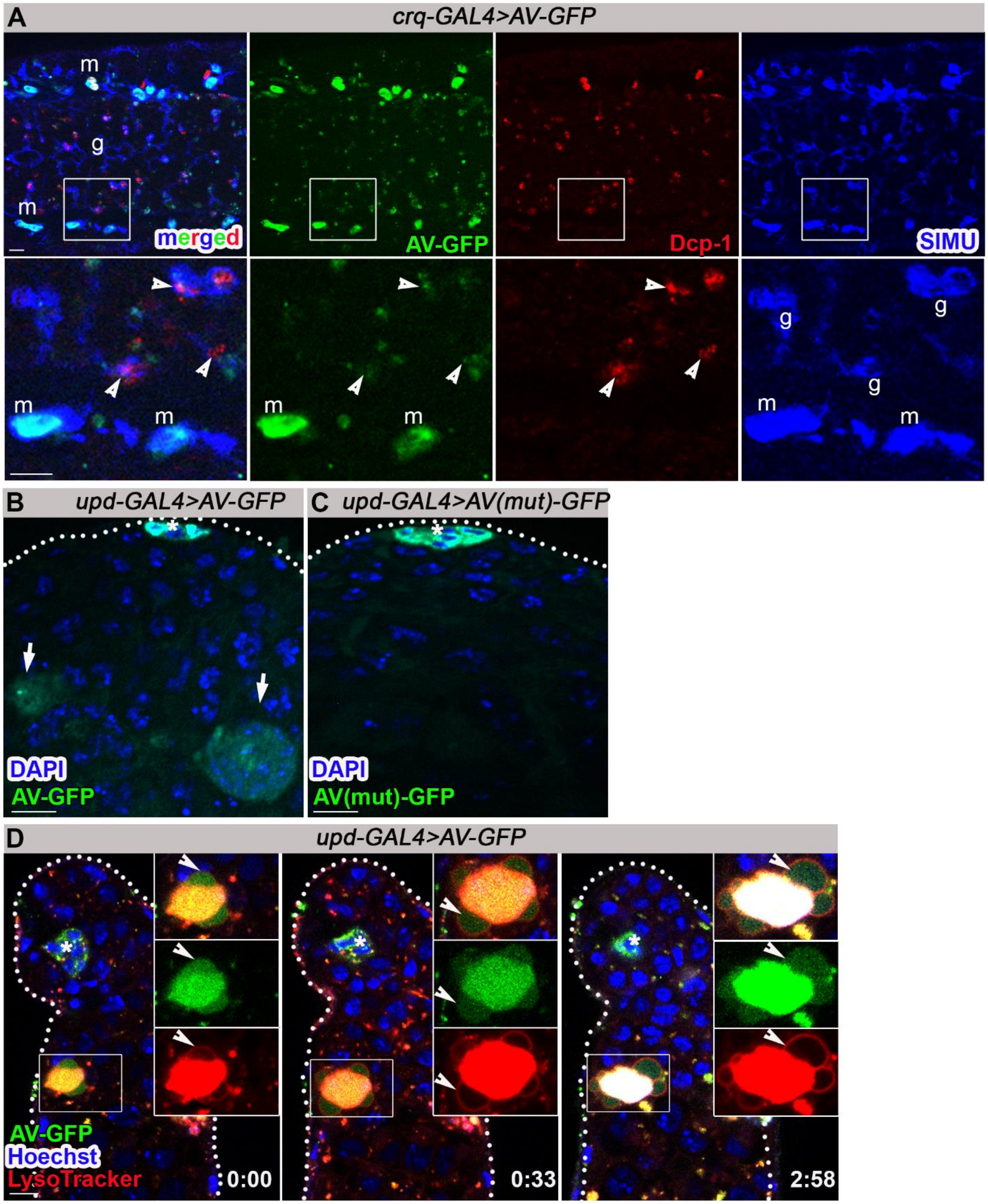

Fig. S6: AV-GFP reporter binds PS on dying germ cells in the testis and on apoptotic neurons in the embryonic CNS. (**A**) Five abdominal segments in whole mount *Drosophila* embryo at stage 16, ventral view, expressing AV-GFP reporter in macrophages (m, *crqGal4/UAS-AV-GFP*). Embryos immunostained for AV-GFP (green), anti-cleaved caspase Dcp-1 to label apoptotic neurons (red), SIMU (blue) to label the membranes of glia (g) and macrophage (m). Macrophages outside the CNS express secreted AV-GFP which is detected on apoptotic cells engulfed by glia (arrowheads). (**B** to **C**) Fixed testes from males expressing AV-GFP (B, green, *updGal4;UAS-AV-GFP*) or AV(mut)-GFP (C, green, *updGal4;UAS-AV(mut)-GFP*) secreted from hub cells. Arrows mark AV-GFP binding to dying germ cells. Note no binding of AV(mut)-GFP. (**D**) Snapshots of live-imaged testis, marked with LysoTracker (red), Hoechst (blue, nuclei) and AV-GFP secreted from hub cells (green, *updGal4;UAS-AV-GFP*). Insets are high magnification views of boxed regions highlighting LysoTracker negative blebs pinching off the dying germ cell, which contain PS. Time (h:min) is shown on the bottom right of the images. Bars represent 10 µm.

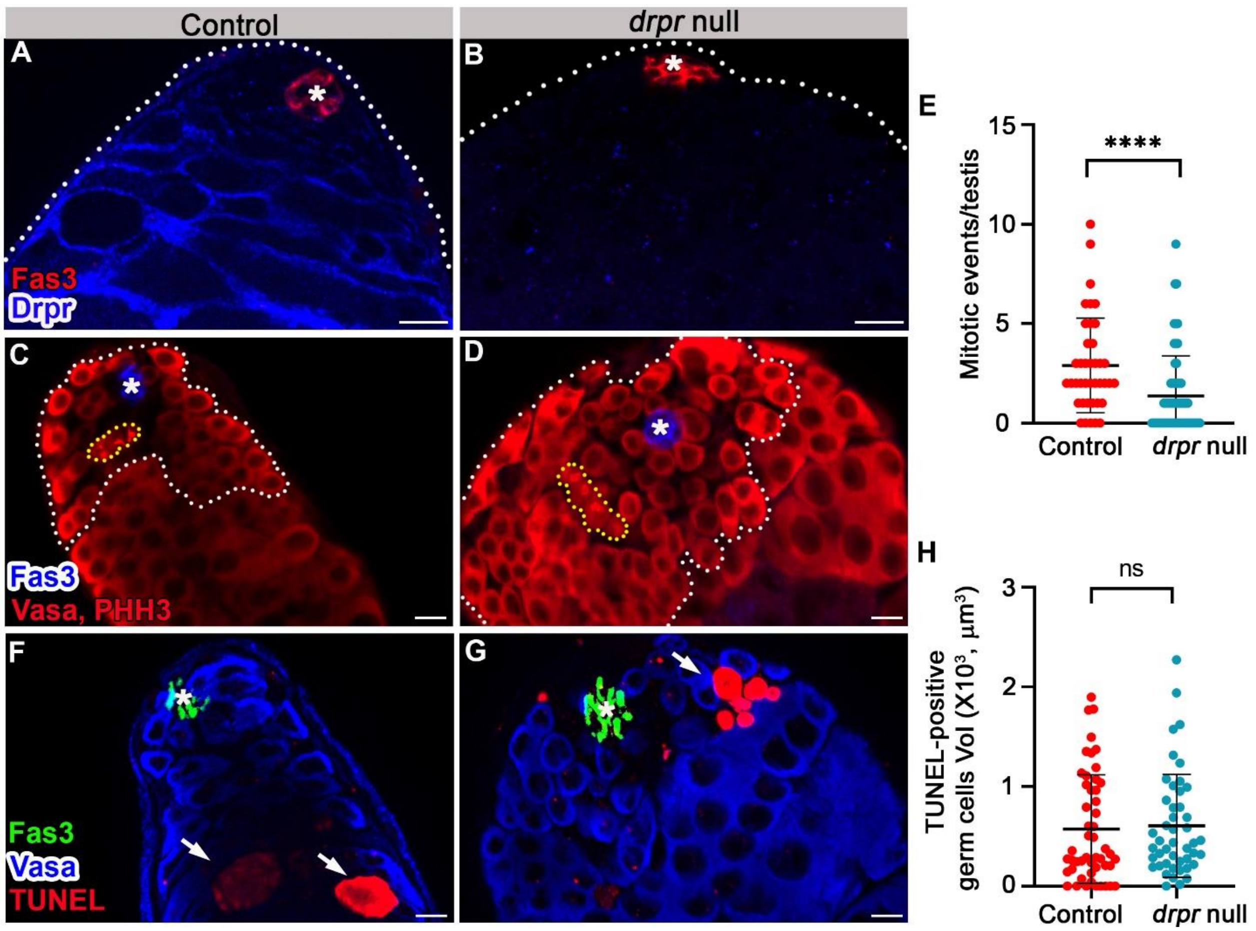

Fig. S7: Drpr is not expressed in cyst cells of *drpr* null and hyperplasia is not due to mitosis. (**A** to **B**) Testes from young (2 day-old) males immunostained for Fas3 (red, hub) and Drpr (blue). (A) Control (*w1118*) and (B) *drpr* null flies. Note that no Drpr signal is detected in cyst cells of *drpr* null flies. The white dashed line delineates the outline of the testes. (**C** to **E**) Testes from 7 day-old males of control (C) and *drpr* null flies (D) immunostained for Fas3 (blue, hub), Vasa (red (cytoplasmic), germ cells) and PHH3 (red (nuclear), mitotic spermatogonia). The white dashed line delineates spermatogonia cells and the yellow dashed line marks dividing spermatogonia. (E) Quantification of PHH3-positive mitotic events in spermatogonia cells of 7 day-old (control, *w1118* (n=40) and *drpr* null males (n=62). (**F** to **H**) Testes from 7 day-old males of control (F) and *drpr* null flies (G) immunostained for Fas3 (green, hub), Vasa (blue, germ cells) and TUNEL (red, dying germ cells). Arrows mark germ cell debris. (H) Quantification of the volume of TUNEL positive germ cells in control (red dots, (n=48)) and *drpr* null (blue dots,

(n=49)) testes as measured with Imaris. Statistical significance was determined by a Mann-Whitney test. **** $p \leq 0.0001$, ns=not significant. Bars represent 10 μm

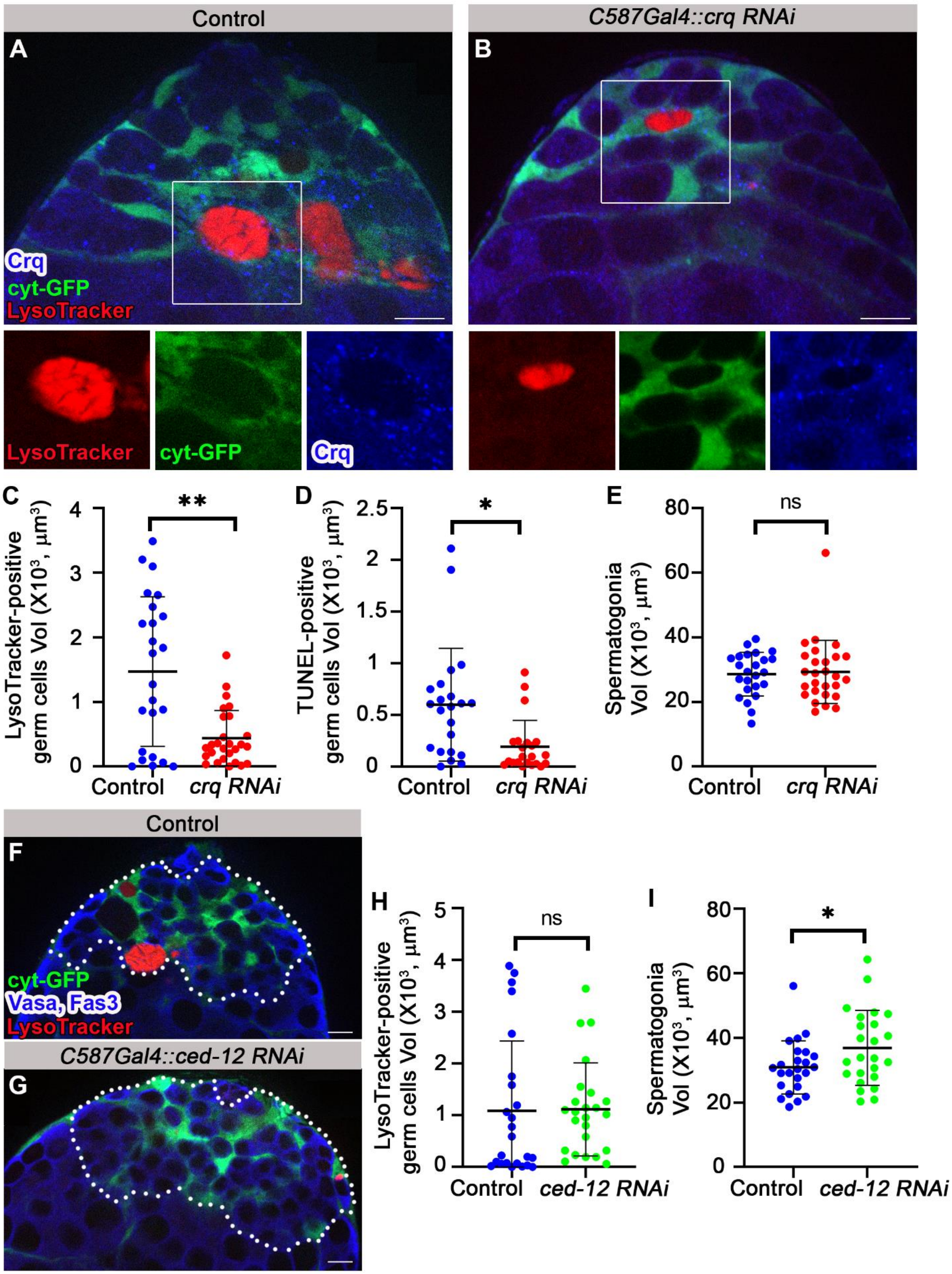

Fig. S8: Crq and Ced12 regulate GCD. (A to **B)** The apical tip of a testes from control flies that expresses GFP in cyst cells (A, green, *c587Gal4;UAS-cytGFP*) and from flies expressing *crq RNAi* in cyst cells (B, *c587-Gal4;UAS-cytGFP,UAS- crq RNAi*), immunostained for Crq (blue) and LysoTracker (red, dying germ cells). Bottom images are high magnification views of the boxed regions, highlighting Crq expression in cyst cells (A) and reduction in *crq RNAi* (B). (**C** to **E**) Quantification of the volume of LysoTracker (C) or TUNEL (D) positive germ cells and live spermatogonia cells (E) as measured with Imaris (control (n=24), blue dots and *crq RNAi* (n=27), red dots). Note significant reduction in GCD in testes of *crq RNAi* and no significant change in spermatogonia volume. (**F** to **I**) Testes of control flies expressing GFP in cyst cells (F, green, *c587Gal4;UAS-cytGFP*) and from flies expressing *ced-12 RNAi* in cyst cells (G, *c587-Gal4;UAS-cytGFP,UAS-ced-12 RNAi*), immunostained for Fas3 and Vasa (blue, hub and germ cells) and LysoTracker (red, dying germ cells). The white dashed line delineates spermatogonia cells. Quantification of the volume of LysoTracker positive germ cells (H) and live spermatogonia cells (I) as measured with Imaris (control (n=24), blue dots and *ced-12 RNAi* (n=24), green dots). Note significant increase in spermatogonia volume and no significant change in GCD in testes of *ced-12 RNAi*. Statistical significance was determined by t-test, $^{**}p \leq 0.01$, $^{*}P \leq 0.05$ and ns=not significant. Scale bars correspond to 10μm.

Movies

Movie S1: Cyst cells marked with cytGFP

A representative example of live, *ex vivo Drosophila* testis of young male is labeled with LysoTracker (red) and Hoechst (blue, nuclei). Scale bars represent 10μm, time (h:min) is shown on the bottom of the movie and single channels are presented in grayscale. Cyst cells are marked with cytGFP (green, *c587Gal4;UAS-cytGFP*). Arrows mark two neighboring GCD events. The first one is completely degraded within a cyst cell in 2h and the second begins after 2h with

packed DNA in separate nuclei that becomes involuted into one bundle. Yellow arrowhead marks LysoTracker positive debris within a cyst cell.

Movie S2: Lamp1-GFP

A live-imaged testis of *Lamp1-GFP* expressed in cyst cells (green, *c587Gal4;UAS- Lamp1-GFP*), labeled with LysoTracker (red). Scale bars represent 10µm, time (h:min) is shown on the bottom of the movie and single channels are presented in grayscale. Arrows mark the fusion of Lamp1-GFP vesicles (after 2h) surrounding live germ cells that are gradually filled with LysoTracker.

Movie S3: Rab7-YFP

Live-imaged testis from Rab7-YFP (green) labeled with LysoTracker (red). Scale bars represent 10µm, time (h:min) is shown on the bottom of the movie and single channels are presented in grayscale. Arrow marks late endosome vesicles surrounding live germ cells (after 3h) that are gradually filled with LysoTracker.

Movie S4: Rab5-YFP

Live-imaged testes from Rab5-YFP (green) flies labeled with LysoTracker (red). Scale bars represent 10µm, time (h:min) is shown on the bottom of the movie and single channels are presented in grayscale. Arrow follows the progression of one GCD event and arrowhead marks the fusion of early endosome vesicles surrounding live germ cells that are gradually filled with LysoTracker.

Movie S5: AV-GFP

3 dimensional projection of live-imaged testis of AV-GFP secreted from hub cells (green, *updGal4;UAS-AV-GFP*) labeled with LysoTracker (red). Scale bars represent 10µm, time (h:min) is shown on the bottom of the movie and single channels are presented in grayscale. Arrowhead marks new GCD event that is only marked by AV-GFP after staining for LysoTracker, and arrows mark 2 GCD events at different stages marked with LysoTracker that progressively expose PS on the germ cell membranes for 4h. Asterisk marks the hub.

Movie S6: AV(mut)-GFP

Live-imaged testis of AV(mut)-GFP secreted from hub cells (green, *updGal4;UAS-AV(mut)-GFP*) labeled with LysoTracker (red) and Hoechst (blue, nuclei). Scale bars represent 10µm, time (h:min) is shown on the bottom of the movie and single channels are presented in grayscale. Arrows mark 2 GCD events at different stages marked with LysoTracker that are not labeled with AV(mut)-GFP. Asterisk marks the hub.

Movie S7: Cyst cells marked with Mem-GFP

Live-imaged testis of a membrane targeted mGFP expressed in cyst cells (green, *c587Gal4;UAS-mGFP*) labeled with LysoTracker (red) and Hoechst (blue, nuclei). Scale bars represent 10µm, time (h:min) is shown on the bottom of the movie and single channels are presented in grayscale. Arrowhead marks a GCD event in which cyst cells' membrane accumulates in DNA degradation domains and recycling vesicles are formed around dying spermatogonia.

Movie S8: TRE-eGFP reporter

Live-imaged testis of TRE-eGFP reporter (green) show signal in all cyst cells labeled with LysoTracker (red) and Hoechst (blue, nuclei). Scale bars represent 10µm, time (h:min) is shown on the bottom of the movie and single channels are presented in grayscale. Arrow marks increased reporter signal around LysoTracker (red) positive germ cells.
